# The landscape of immune checkpoint-related long non-coding RNAs core regulatory circuitry reveals implications for immunoregulation and immunotherapy responses

**DOI:** 10.1038/s42003-024-06004-z

**Published:** 2024-03-14

**Authors:** Changfan Qu, Hao Cui, Song Xiao, Longlong Dong, Qianyi Lu, Lei Zhang, Peng Wang, Mengyu Xin, Hui Zhi, Chenyu Liu, Shangwei Ning, Yue Gao

**Affiliations:** 1 College of Bioinformatics Science and Technology, Harbin Medical University, Harbin, 150081 China; 2 The Second Affiliated Hospital of Harbin Medical University, Harbin, China

**Keywords:** Computational biology and bioinformatics, Immunology

## Abstract

Long non-coding RNAs (lncRNAs) could modulate expression of immune checkpoints (ICPs) by cooperating with immunity genes in tumor immunization. However, precise functions in immunity and potential for predicting ICP inhibitors (ICI) response have been described for only a few lncRNAs. Here we present an integrated framework that leverages network-based analyses and Bayesian network inference to identify the regulated relationships including lncRNA, ICP and immunity genes as ICP-related LncRNAs mediated Core Regulatory Circuitry Triplets (ICP-LncCRCTs) that can make robust predictions. Hub ICP-related lncRNAs such as MIR155HG and ADAMTS9-AS2 were highlighted to play central roles in immune regulation. Specific ICP-related lncRNAs could distinguish cancer subtypes. Moreover, the ICP-related lncRNAs are likely to significantly correlated with immune cell infiltration, MHC, CYT. Some ICP-LncCRCTs such as CXCL10-MIR155HG-ICOS could better predict one-, three- and five-year prognosis compared to single molecule in melanoma. We also validated that some ICP-LncCRCTs could effectively predict ICI-response using three kinds of machine learning algorithms follow five independent datasets. Specially, combining ICP-LncCRCTs with the tumor mutation burden (TMB) improves the prediction of ICI-treated melanoma patients. Altogether, this study will improve our grasp of lncRNA functions and accelerating discovery of lncRNA-based biomarkers in ICI treatment.

## Abstract (web summary)

An integrated framework to identify immune checkpoint-related lncRNA core regulatory circuitry triplets has the potential to improve our understanding of lncRNA functions and accelerate the discovery of lncRNA-based biomarkers for immune checkpoint inhibitor treatment.

## Introduction

Immune checkpoint inhibitors (ICIs) activate immune cells by inhibiting immune checkpoints (ICPs), thus blocking the immune escape of tumor cells. ICIs have successfully changed the treatment prospects for many cancer types, especially melanoma and lung cancer^1^. Several pivotal trials have shown that ICIs, as either monotherapies or combination therapies, improve most clinical efficacy endpoints for patients with locally advanced or metastatic cancers^2^. Although ICIs can provide clinical benefit, there are some main limitations which include low response rates, complex and diverse immune-related adverse events (irAEs), and varying degrees of drug resistance. Therefore, it is urgent to address the problems in current cancer research involving ICIs requires studying the regulatory mechanism of ICP-related genes at multiple molecular levels, such as protein-coding genes and non-coding genes, predicting the therapeutic efficacy of ICIs, and identifying early predictive biomarkers of ICI efficacy to terminate ineffective treatment as soon as possible.

There is a large amount of non-coding RNA in the human genome, which is widely involved in the occurrence and development of a variety of cancer types and the effect of drug therapy^3^. Long-chain non-coding RNAs (lncRNAs) are a very important class of non-coding RNAs with a length of more than 200 bases that play an essential role in the process of cancer progression^4,5^. In particular, during the regulation of the tumor-immune response, lncRNAs can play an essential role by regulating necessary immune genes and pathways. With continuous in-depth research in recent years, the mechanisms and functions of several lncRNAs in cancer have become increasingly clear. For example, Joseph Toker et al. suggested an association between NEAT1 expression and patient response to anti-PD-1/PD-L1 therapy in melanoma and glioblastoma^6^. However, the regulatory mechanisms and functional effects of most lncRNAs and ICP molecules are still unknown.

Innate and adaptive immunity are strongly dependent on a series of mRNA-based regulatory events. LncRNAs are a kind of important gene regulatory factor of these processes^7^. LncRNAs are emerging as critical regulators of gene expression in the immune system^8^. For example, the lncRNA SATB2-AS1 was downregulated in colon cancer and can inhibit tumor metastasis by regulating the gene encoding STAB2 and affecting the tumor-immune microenvironment^9^. An increasing number of studies have shown that lncRNAs can directly or indirectly regulate ICP molecules, leading to heterogeneity in ICI efficacy and the development of drug resistance^10^. For example, in diffuse large B-cell lymphoma, the lncRNA SNHG14 upregulates the mRNA ZEB1 by competitively binding to miR-5590-3p, and ZEB1 positively activates SNHG14 and PD-L1, thereby promoting immune escape of tumor cells^11^. Thus, the core regulatory circuitry consisting of lncRNAs, immune-related mRNAs, and ICP genes is crucial for exploring the regulatory mechanisms of ICP molecules in tumors.

In this study, we report an integrated framework that leverages network-based analyses and Bayesian network inference to (i) identify ICP-related lncRNAs and (ii) infer the regulatory patterns of ICP-LncCRCTs. The identified results were validated in independent datasets and databases. Four basic regulatory patterns were inferred by the Bayesian network and maximum likelihood estimation. Immunity-related genes play crucial roles in the process through which lncRNAs regulate ICPs. Common and specific ICP-related lncRNAs participate in diverse immune processes. Cancer-specific ICP-related lncRNAs can distinguish cancer types. ICP-related lncRNAs were correlated with immune cell infiltration in cancers based on bulk data, immune cell lines, and scRNA-seq datasets. Some ICP-LncCRCTs could better predict patient prognosis than single molecules in melanoma. We also validated that several ICP-LncCRCTs could effectively predict ICI response using three kinds of machine learning algorithms and five independent datasets. Specifically, combining ICP-LncCRCTs with the tumor mutation burden (TMB) improved the prediction of ICI-treated melanoma patients. In summary, our method provides an approach to unveil insights into lncRNAs regulating ICPs in tumor immunity, helping previously identified biomarkers improve the prediction of ICI response.

## Results

### Identification of ICP-related lncRNA-mediated core regulatory circuitry triplets (ICP-LncCRCTs) across cancer types

**Fig. 1:**
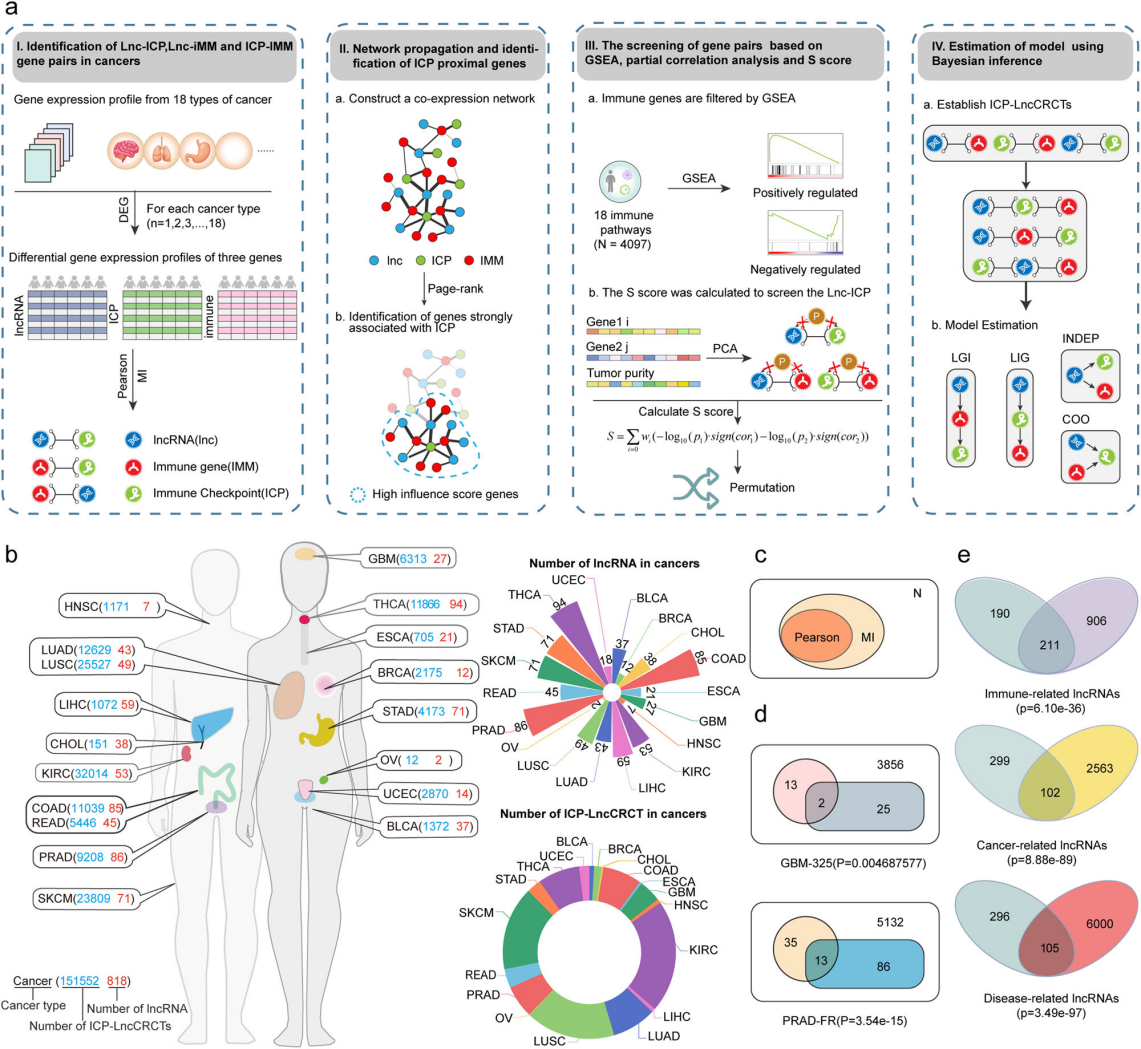
The computational algorithm for identification of ICP-LncCRCTs. **a** Schematic representation of the four steps in the identification of ICP-LncCRCTs. **b** The number of ICP-related lncRNAs and ICP-LncCRCTs in each cancer type. **c** Venn diagram showing the overlap of coexpressed gene pairs detected using the PCC and MI methods. The hypergeometric test was used to assess the significance of the overlap. **d** Venn diagram of the results identified with other independent datasets. The intersections represent jointly identified results. The hypergeometric test was used to assess the significance of the overlap. **e** Venn diagram of ICP-related lncRNAs and immune-, cancer- and disease-related lncRNAs. The intersection represents the common lncRNAs. The hypergeometric test was used to assess the significance of the overlap.

To identify candidate lncRNAs that can drive ICPs by forming core regulatory circuitry triplets, we proposed a four-step computational framework (Fig. 1a). The framework systematically infers ICP-related lncRNAs and their regulatory mechanisms from expression profiles, network modules, and immune-related pathways based on a large number of samples. We presumed that if lncRNAs could participate in immunology by regulating ICPs, then they closely interact with immune genes and ICPs and could also be enriched in immune-related pathways. Overall, four steps were applied to identify ICP-LncCRCTs. First, differentially expressed immune gene, lncRNA, and ICP for cancer patients were extracted. Second, genes that were strongly correlated with each other were screened via Pearson correlation analysis to construct co-expression networks. We used the page-rank algorithm to identify the immune genes and lncRNAs that were closely associated with ICP. Third, based on excluding the influence of tumor purity using the partial correlation analysis, GSEA, S score, and permutation, we further identified lncRNAs, immune genes, and ICP genes that might have a ternary regulatory association (named ICP-LncCRCT). Finally, we inferred the best regulatory pattern for each ICP-LncCRCT using Bayesian inference.

By applying the four-step computational framework, ICP-related lncRNAs and ICP-LncCRCTs were extracted from diverse cancer types (Fig. 1b). The numbers of ICP-related lncRNAs and ICP-LncCRCTs exhibited a greater range (2–94 and 12–25,527) by excluding the influence of sample size. Although a moderate number of ICP-related lncRNAs were identified, most of the identified ICP-LncCRCTs were associated with SKCM and LUSC. These findings indicated that lncRNAs could play complex roles in immunology by forming a multitude of ICP-LncCRCTs in cancers. The reliability of the framework was validated in three parts. First, the associations among lncRNAs, the ICP and immune genes were significantly intersected according to two methods, PCC and MI (Fig. 1c). Second, similar results were also validated in other independent datasets (GBM and PRAD) after removing batch effects (Fig. 1d, Supplementary Fig. 1). Finally, we found that the ICP-related lncRNAs had significant interactions with immune-, cancer- and disease-related lncRNAs (Fig. 1e). Taken together, these results revealed that ICP-LncCRCTs could be identified and considered valuable resources in cancer immunology.

### ICP-LncCRCTs involve complex regulatory patterns across cancers

**Fig. 2:**
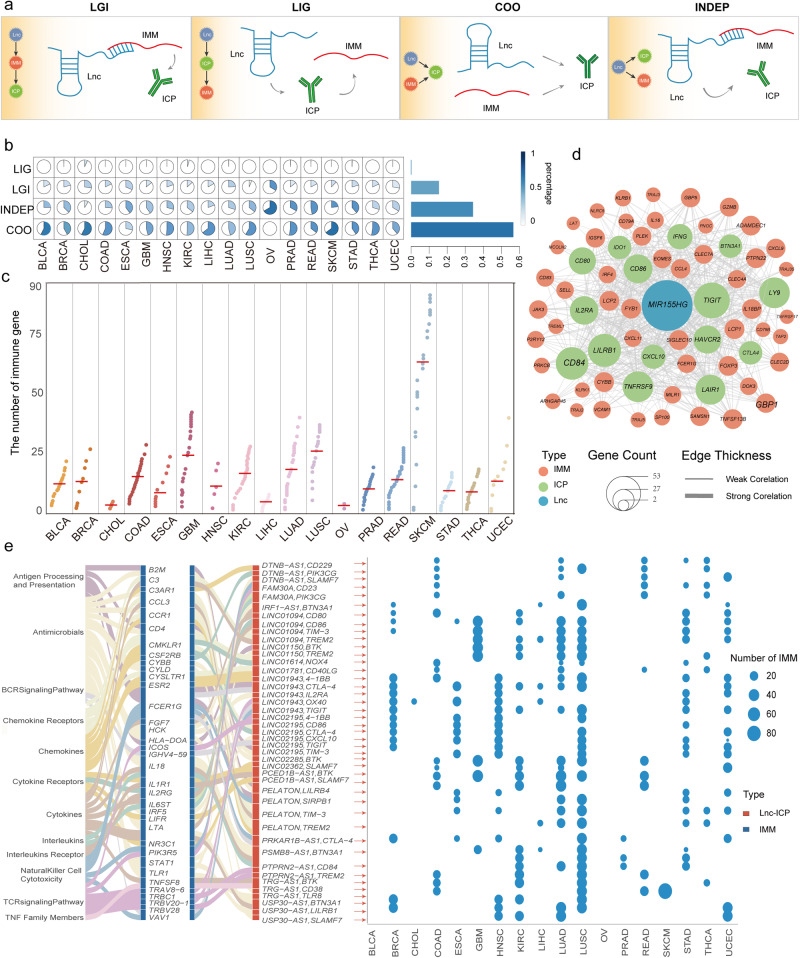
ICP-LncCRCTs have complex regulatory mechanisms. **a** Four possible regulatory patterns. **b** The proportions of the four regulatory patterns in all cancers. **c** The numbers of immune genes comprising ICP-LncCRCTs in each cancer type. Different colors indicate different types of cancer. The red horizontal line indicates the mean value. **d** Subnetwork of ICP-LncCRCTs, including MIR155HG. Red, green, and blue indicate the immune gene, ICP gene, and lncRNA, respectively. The size of the nodes indicates the number of connected nodes. The thickness of the edges indicates the level of correlation. **e** The same lncRNA-ICP gene pair can interact with different ICP-LncCRCTs via different immune genes involved in diverse immune pathways. The blue bubble diagram indicates the number of immune genes affected by each lncRNA-ICP gene pair in each cancer. A Sankey diagram indicated that each lncRNA-ICP gene pair participates in different immune pathways by mediating different immune genes. The first column of the Sankey diagram indicates the different immune pathways, and the second column indicates the immune genes. The third column indicates the lncRNA-ICP gene pairs.

To evaluate the mechanism through which lncRNAs regulate ICPs, four major regulatory patterns of ICP-LncCRCTs were proposed to elucidate the possible relationships within a triplet. The four regulatory mechanisms were named lncRNA-mediated regulation of ICP by immune genes (LGI) and lncRNA-mediated direct regulation of ICP, further influencing immune genes (LIGs), coordinates (COOs), and independence (INDEP). In the “Independent” pattern, lncRNAs acted as an independent regulator modulating ICP and immunity gene expression. In the “Coordinate” pattern, lncRNAs and immunity genes could act as synergistic regulators to affect the expression of the target ICP genes (Fig. 2a). The frequency of the four kinds of regulatory patterns varied across cancer types. In general, the COO and LIG regulatory patterns had the largest and the smallest proportions, respectively, across cancers (Fig. 2b). Thus, immune genes produce a marked effect on the process through which lncRNAs regulate ICPs. Almost all the ICP-LncCRCTs were formed by multiple immune genes (Fig. 2c, Supplementary Fig. 2). For example, the ICP-related lncRNA MIR155HG could regulate ICP gene CXCL10 by binding to the ICOS immune gene via the ICP-LncCRCT complex (Fig. 2d). Many studies have reported that MIR155HG is associated with immune infiltration and the expression of immune checkpoint molecules in a variety of cancers^12–14^. We also found that the immune regulatory effects of the identified ICP-related lncRNAs were mediated by different numbers of immune genes involved in regulating ICP in different cancers. In addition, an identical lncRNA-ICP gene pair could form different ICP-LncCRCTs with different immunity genes and participate in diverse immune pathways to perform their functions. (Fig. 2e). In summary, these results indicated that ICP-LncCRCTs involve complex regulatory patterns across cancers.

### Common and specific ICP-related lncRNAs play diverse roles in immune-related cancers

**Fig. 3:**
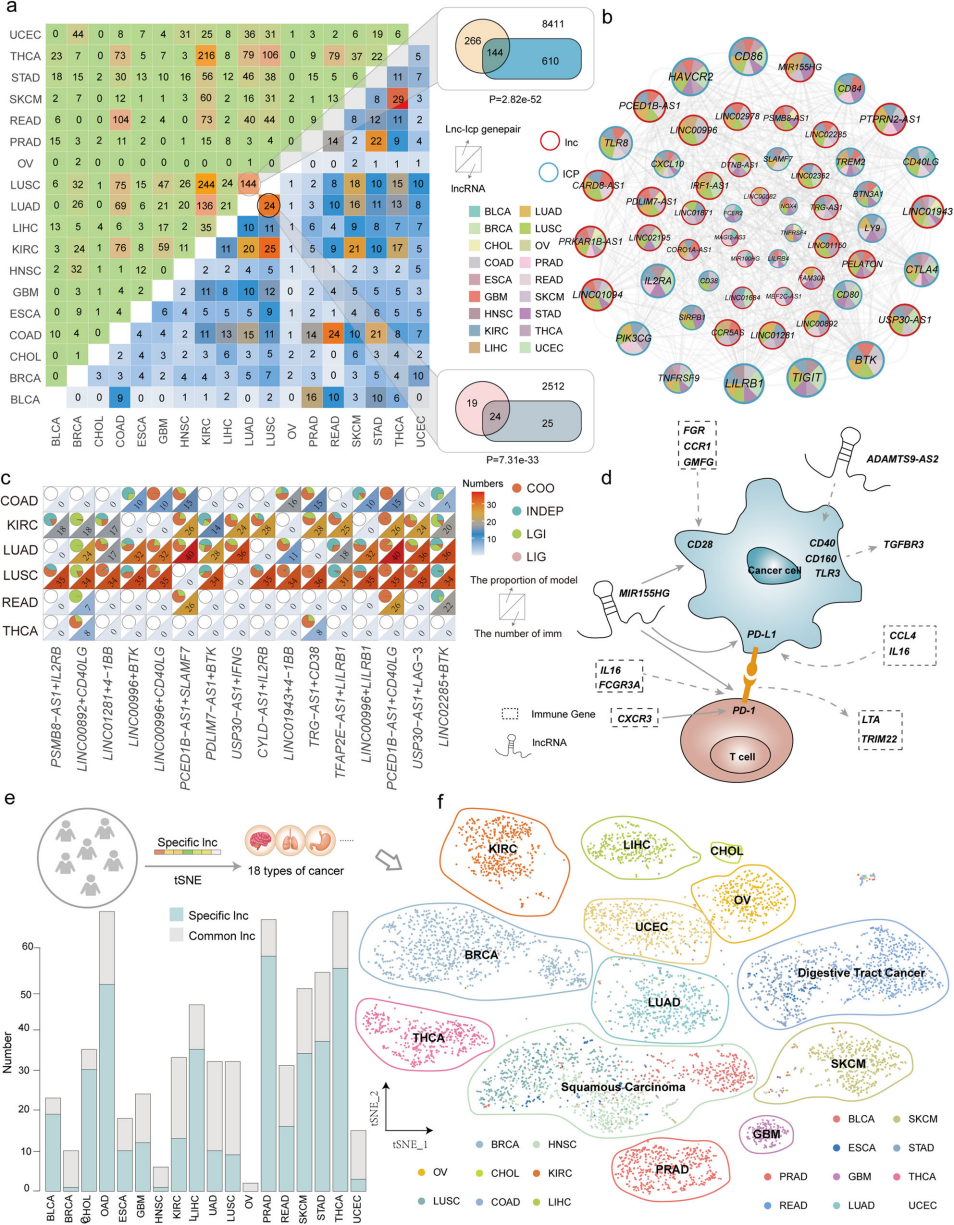
Common and specific ICP-related lncRNAs exhibited diverse immune regulation patterns. **a** The upper triangular matrix shows the number of shared lncRNA-ICP gene pairs between any two cancers. The lower triangular matrix shows the number of shared ICP-related lncRNAs. A Venn diagram represents the overlap of lncRNA-ICP gene pairs as well as ICP-related lncRNAs in two lung cancer subtypes, LUAD and LUSC. The hypergeometric test was used to assess the significance of the overlap, and the *p* values are shown below the figure. **b** Common lncRNA network. The blue and red nodes indicate the ICP and common lncRNAs, respectively. Pie charts of different colors indicate the presence of genes in different cancers. The size of the nodes indicates the number of connected nodes. The thickness of the edges indicates the level of correlation. **c** Patterns and numbers of regulated immune genes among the pattern-variable lncRNA-ICP gene pairs in different cancers. The pie chart represents the percentages of the four models. The heatmap represents the number of immune genes. **d** Inferred mechanisms by which ICP-LncCRCTs act between immune cells and tumor cells. **e** Stacked bars indicate the number of common lncRNAs and specific lncRNAs in each cancer. **f** Cancer samples were clustered by t-distributed stochastic neighbor embedding (t-SNE) based on specific lncRNAs. Different colors represent different cancer types.

To further investigate the crucial roles of ICP-related lncRNAs in diverse cancer types, ICP-LncCRCTs were characterized in each cancer type. For all lncRNAs in the ICP-LncCRCTs, lncRNAs identified in five or more types of cancer were defined as common lncRNAs, and lncRNAs identified in two or fewer types of cancer were defined as specific lncRNAs. We chose “five” to define common lncRNAs because the numbers of common lncRNAs were similar when five to other (6~14) were changed (Supplementary Fig. 3). Subtypes with similar tissue-of-origin characteristics shared common ICP-related lncRNAs and ICP-LncCRCTs (Fig. 3a). For example, the similarity of ICP-related lncRNAs and ICP and lncRNA pairs was greater among two subtypes of lung cancer, LUAD and LUSC. More than 50% of the ICP and lncRNA pairs in LUAD also occurred in LUSC, which was significant according to Fisher’s test (*P* = 2.34e-43). Similar results were also found in COAD and READ, which are two intestinal cancer types (lncRNA: *P* = 2.69e-19; ICP and lncRNA pairs: *P* = 5.96e-58). The ICP-LncCRCT networks were constructed and analyzed for each cancer type. The degrees of the lncRNAs were greater than those of the ICPs and immune genes in all cancer types, which indicated that the regulatory association may be ICP-centered in most cancer types (Supplementary Fig. 4). Several ICP-related lncRNAs can be found in many kinds of cancer. These common lncRNAs exhibit close interactions with ICP genes in many cancers (Fig. 3b). In this common lncRNA network, several ICP-related lncRNAs and genes have been validated in other studies. For example, MIR155HG can regulate the tumor-immune microenvironment through cytokine‒cytokine receptor interactions and complement and coagulation cascades and is positively correlated with TIM-3 expression^15^. PSMB8-AS1 promotes pancreatic cancer progression by regulating the miR-382-3p/STAT1/PD-L1 axis^16^.

In addition to common lncRNAs, several ICP-lncRNA pairs have been identified in multiple cancer types. Although common ICP-lncRNA pairs are present in multiple cancer types, the immune-related genes and regulatory mechanisms involved are diverse across cancer types. The vast majority of the ICP-lncRNA pairs were mediated by 20 to 40 immune genes and more than two kinds of regulatory mechanisms in different cancer types (Fig. 3c). For example, PSMB8-AS1 is a common ICP-related lncRNA, and multiple immune genes that mediate and exhibit different regulatory patterns can be found in gene pairs composed of PSMB8-AS1 and IL2RB. We referred to this class of gene pairs as pattern-variable lncRNA-ICP relationship pairs (Fig. 3c). The common ICP-related lncRNA MIR155HG is involved in the interaction of ICP-LncCRCTs with the ICP genes PD1, PDL-1 and CD28, which are mediated by six different immunity genes in tumors and T cells (Fig. 3d). Some of these regulatory associations have been verified in previous studies^13,17^. Taken together, these results indicated that the regulatory associations between lncRNAs and ICP are complex.

Specific ICP-related lncRNAs were also characterized. The number of specific ICP-related lncRNAs was diverse (from 0 to 55) across cancer types (Fig. 3e). These expression patterns of specific ICP-related lncRNAs were further confirmed by t-distributed stochastic neighbor embedding (t-SNE) (Fig. 3f). Diverse cancer types could be distinguished, and cancers with related tissue origins or the same cancer type were clustered together, exhibiting similar lncRNA expression patterns, including core gastrointestinal cancer (ESCA, STAD, COAD and READ) and squamous cell carcinoma (LUSC, HNSC). Taken together, these findings revealed that ICP-related lncRNAs exhibit common and specific characteristics in cancer, suggesting that they play diverse roles in oncogenic processes and the tumor-immune microenvironment.

### ICP-related lncRNAs were correlated with immune cell infiltration in cancers

**Fig. 4:**
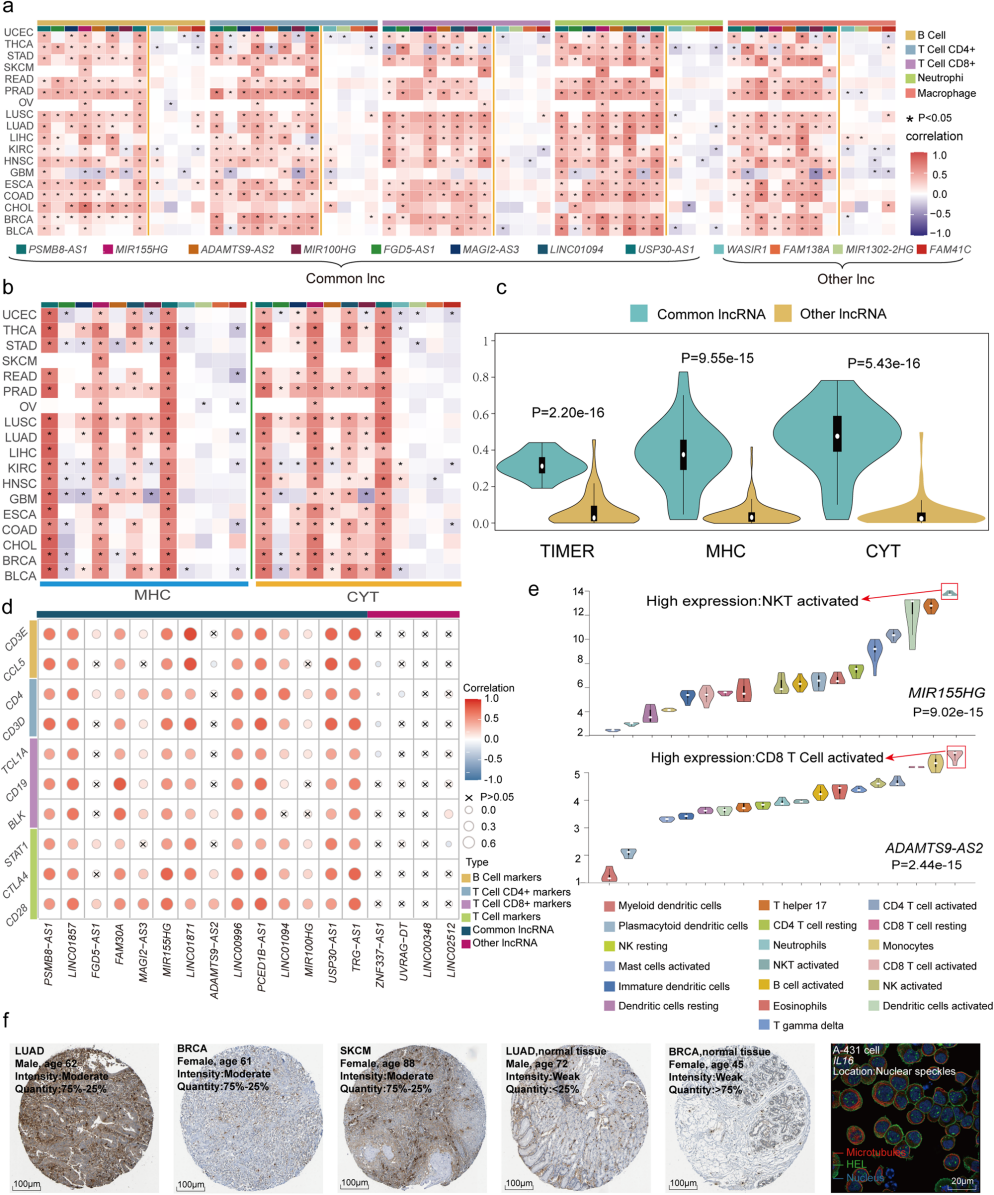
Relationship between ICP-related lncRNAs and immune system activity. **a** Heatmap showing the correlation of 18 common lncRNAs and specific lncRNAs with TIMER scores. “*” indicates a strong correlation. **b** Heatmap representing the correlation of common lncRNAs and specific lncRNAs with the MHC score and CYT score. **c** Violin plots representing the overall comparison of common lncRNAs and specific lncRNAs with MHC scores and CYT scores. Black boxes indicate the interquartile range of the data. White dots indicate the median. Black vertical lines indicate 95% confidence intervals. The width of the violin plot indicates the density of the data. **d** Correlations between common lncRNAs and specific lncRNAs and markers of four classes of immune cells. The size of the circle and the shade of the color indicate the magnitude of the correlation. “x” indicates no correlation (*P* > 0.05, Pearson correlation analysis). **e** Violin plots indicating the differential expression of MIR155HG and ADAMTS9-AS2 in 19 immune cell lines (Kruskal‒Wallis test; *p* values are displayed at the bottom of the plots). Black boxes indicate the interquartile range of the data. White dots indicate the median. Black vertical lines indicate 95% confidence intervals. The width of the violin plot indicates the density of the data. **f** Immunohistochemistry (IHC) staining of C3AR1 in LUAD, BRCA, and SKCM tissues. Immunofluorescence (IF) staining of IL16 in A-431 cells.

The immune response has been proven to involve tissue infiltration of immune cells. Therefore, we hypothesized that if ICP-related lncRNAs could perform essential and important functions in immune regulation, they would tend to be highly expressed in immune cells and associated with immune cell infiltration in tumors. Immune cell infiltration levels were estimated by TIMER based on lncRNA expression profiles. Five kinds of tumor-infiltrating immune cells, B cells, CD4 T cells, CD8 T cells, macrophages, and neutrophils, were analyzed. A large number of ICP-related lncRNAs were correlated with immune cell infiltration (Fig. 4a, Supplementary Fig. 5). In particular, common ICP-related lncRNAs were more related to immune cell infiltration than other ICP-related lncRNAs were (Supplementary Fig. 6). In addition, the MHC and CYT scores were greater for common lncRNAs than for specific lncRNAs (Fig. 4b, c). Similar results were also found for immune cell markers. These common ICP-related lncRNAs were also more positively correlated with immune cell markers, including B cells, CD4+ T cells, CD8+ T cells, and T cells, than were the other lncRNAs (Fig. 4d). Then, we analyzed the expression profiles of 19 immune cell lines. The expression of 65% ICP-related lncRNAs, such as MIR155HG and ADAMTS9-AS2, was significantly up-regulated in the T-cell subset (Fig. 4e, Supplementary Fig. 7). In addition, several genes related to ICP-lncRNAs among the ICP-LncCRCTs could also be related to immune regulation. We observed that immune genes in ICP-LncCRCTs had greater protein intensity in cancer tissues than in normal tissues by immunohistochemical (IHC) and immunofluorescent (IF) staining. For example, the C3AR1 gene and HEL gene were affected (Fig. 4f). In summary, ICP-related lncRNAs are likely associated with immune cell infiltration, further validating the results of our algorithm.

### ICP-related lncRNAs showed specific features across immune cell subsets based on scRNA-seq data

**Fig. 5:**
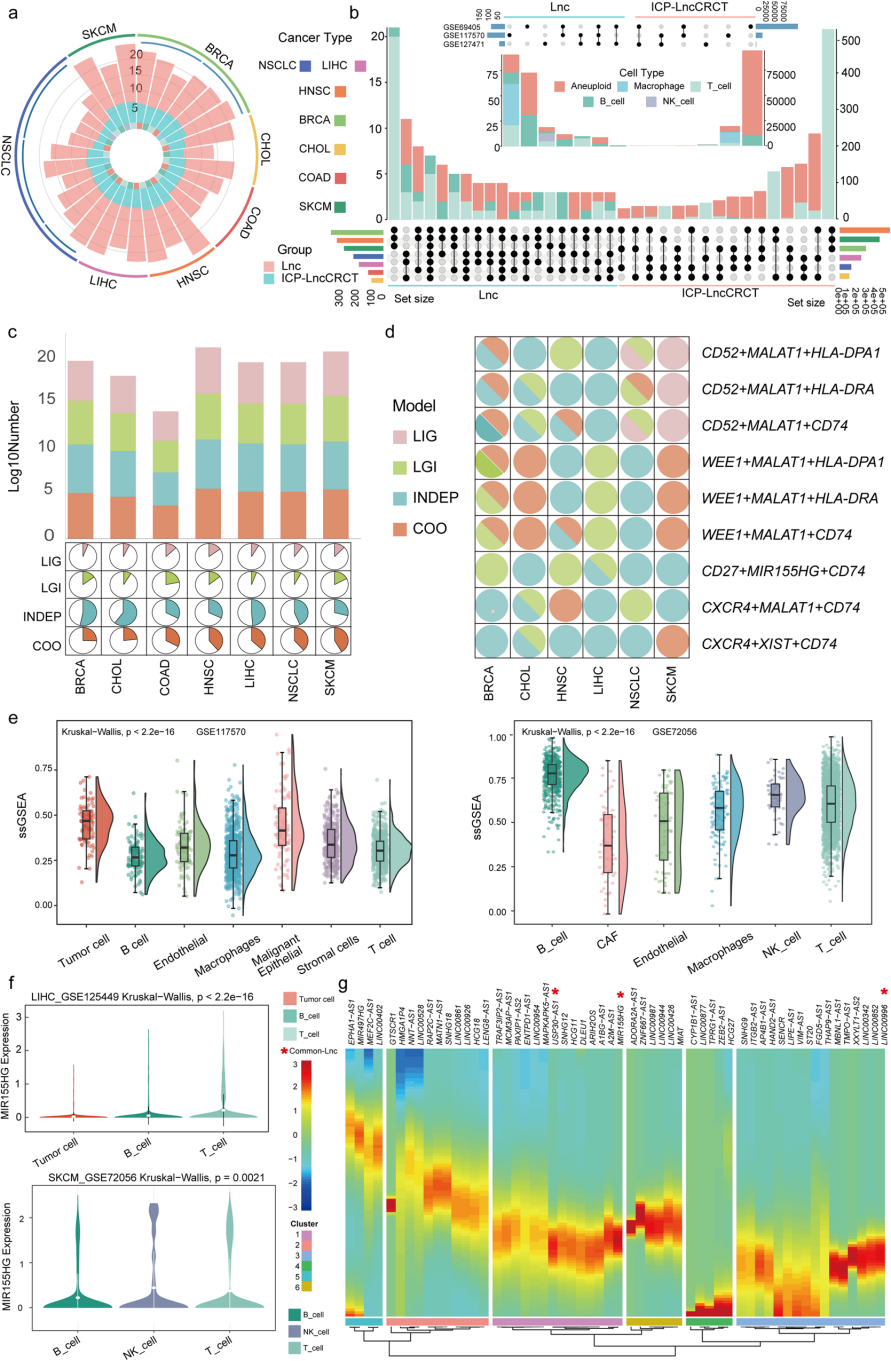
ICP-related lncRNAs are specific to immune cell subsets according to the scRNA-seq data. **a** Quantitative characterization of ICP-associated lncRNAs and ICP-LncCRCTs in single-cell datasets. **b** Upset plots for all single-cell datasets and the lung cancer single-cell dataset. The intersection of ICP-associated lncRNA and ICP-LncCRCTs between the different datasets is shown. The bar graph on the left side shows the total number contained in each dataset. The lower dot plot shows the intersection relationship between each dataset, where each dot represents a dataset and connected dots indicate datasets with intersection. The upper bar graph shows the number of each intersection case. **c** Pie charts and bar graphs show the percentage of the four patterns in the single-cell dataset for different cancers. **d** Pie charts representing ICP-LncCRCTs share of the four modalities in different cancer single-cell datasets. **e** Comparison of ssGSEA scores between different cell types in the GSE117570 and GSE72056 datasets. *P* values were calculated using Kruskal–Wallis. The horizontal lines of the box plot represent the maximum, upper quartile, median, lower quartile, and minimum values. The width of the kernel density plot represents the density of the data. **f** Violins indicate the differential expression of MIR155HG in different types of immune cells in the GSE125449 and GSE72056 datasets. White dots indicate the median. Black vertical lines indicate 95% confidence intervals. The width of the violin plot indicates the density of the data. **g** Pseudotime diagram of ICP-related lncRNAs. “*” indicates lncRNAs that are common ICP-related lncRNAs in genes clustered with MIR155HG.

We further analyzed the scRNA-seq data to explore the roles of ICP-related lncRNAs in diverse immune cell subsets. The ten scRNA-seq datasets, including seven cancer types, were collected and analyzed. The major cell types included tumor cells, B cells, macrophages, NK cells, and T cells (Figure S8). The numbers of ICP-LncCRCTs and ICP-related lncRNAs in diverse cell types were cancer-specific (Fig. 5a). More ICP-LncCRCTs were identified in T cells for the vast majority of cancer types. Like in tissue, there were some common shared ICP-LncCRCTs and ICP-related lncRNAs among diverse cancer types (Fig. 5b). Specifically, seven ICP-related lncRNAs were found in T and B cells for all cancer types. We could infer that ICP-related lncRNAs were relatively stable in T and B cells. For individual datasets from common cancers, more than half of the ICP-LncCRCTs and ICP-related lncRNAs were present in only one dataset (Fig. 5c). These findings indicated that the tumor microenvironment has strong heterogeneity. Like the regulatory patterns of ICP-LncCRCTs in tissue, we found that the COO and INDEP regulatory patterns accounted for the largest proportion of the pancancer cohort (Fig. 5d). The ssGSEA revealed that the scores of ICP-related lncRNAs were greater for T cells, B cells and NK cells (Fig. 5e). For example, the ICP-related lncRNA MIR155HG, which is also a common lncRNA, was differentially expressed in diverse kinds of immune cell types, especially in T cells (Fig. 5f, Supplementary Fig. 8). Consistent results were also shown in the above analysis based on immune infiltration and cell lines. In addition, the associations between ICPs and lncRNAs in ICP-LncCRCTs were also closer in T cells for several cancer types (Supplementary Fig. 9). These ICP-related lncRNAs were dynamically changed at diverse pseudotime points (Fig. 5g). Common ICP-related lncRNAs USP30-AS1, could cluster with MIR155HG and exhibited close co-expression (Supplementary Fig. 10). Collectively, these results indicated that ICP-related lncRNAs could play a role in performing the functions of T cells, B cells and NK cells, further validating their roles in the tumor-immune microenvironment.

### Several ICP-LncCRCTs and ICP-related lncRNAs are associated with survival, and the MIR155HG/CXCL10/EBI3 axis can predict the prognosis in SKCM

**Fig. 6:**
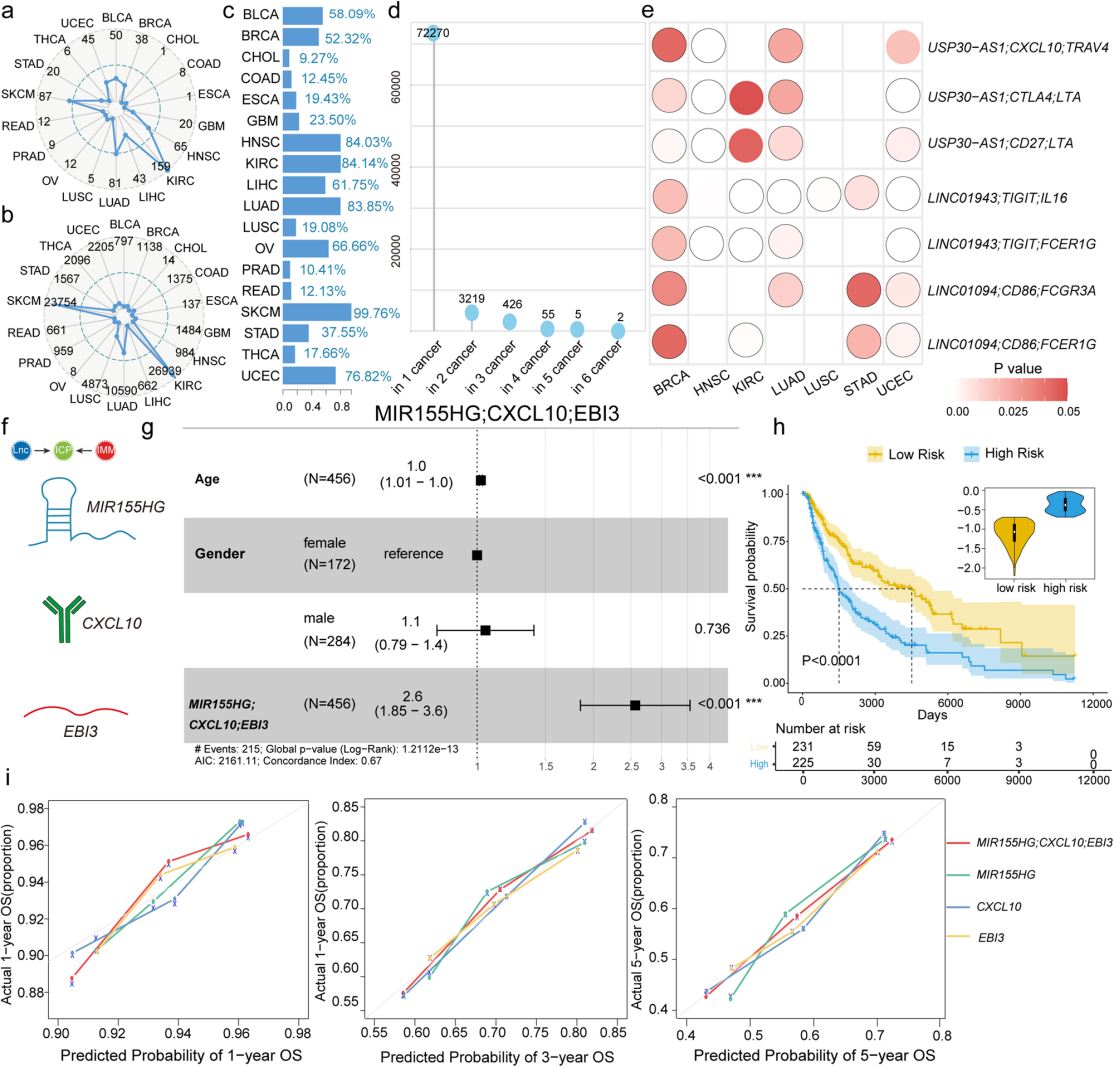
ICP-LncCRCTs are associated with cancer prognosis. **a** Radar plot representing the number of prognostic ICP-related lncRNAs in each type of cancer. **b** The radar plot represents the number of prognostically relevant ICP-LncCRCTs in each type of cancer. **c** Survival-related ICP-LncCRCTs as a percentage of total ICP-LncCRCTs in each cancer type. **d** Lollipop chart showing the numbers of cancer types in which the same survival-related ICP-LncCRCTs were found. **e** Nine ICP-LncCRCTs can be used as prognostic biomarkers in five cancer types. **f** Regulatory mechanism of MIR155HG/CXCL10/EBI3. **g** Forest plot of the MIR155HG/CXCL10/EBI3 risk score. The dashed line is the null line, indicating OR = 1. Each horizontal line indicates a 95% confidence interval. Squares indicate point estimates. **h** KM curves of the MIR155HG/CXCL10/EBI3-related genes in the high- and low-risk groups. Solid blue lines indicate high-risk groups, and solid yellow lines indicate low-risk groups. Shaded areas indicate 95% confidence intervals. Asterisks on the curves indicate censoring points. **i** The ability of MIR155HG/CXCL10/EBI3 to predict 1-, 3- and 5-year OS versus a single gene.

Numerous studies have elucidated how various components of the immune system control or contribute to cancer progression, thus revealing their prognostic value^18^. We considered whether these ICP-related lncRNAs, especially ICP-LncCRCTs, were associated with the survival of cancer patients. A large number of ICP-related lncRNAs and ICP-LncCRCTs were associated with survival in patients with diverse cancers (Fig. 6a, b; Supplementary Fig. 11). Especially in SKCM, 99.76% ICP-LncCRCTs were related to survival (Fig. 6c). The most survival-related ICP-LncCRCTs were found in SKCM, LUAD and KIRC patients who had been treated with ICIs in the clinic. Most of the ICP-LncCRCTs were cancer-specific and were associated with prognosis in only one or two cancer types (Fig. 6d). However, seven ICP-LncCRCTs could serve as prognostic biomarkers in four kinds of cancer (Fig. 6e). We found that USP30-AS1 and LINC01943 were two key ICP-related lncRNAs that participate in multiple survival-related ICP-LncCRCTs with different ICPs and immune genes in diverse cancers. Previously, USP30-AS1 was reported to mediate the progression of various human diseases, such as colon cancer, glioblastoma, cervical cancer, and acute myeloid leukemia^19,20^. According to our above analysis, MIR155HG was the key common ICP-related lncRNA. According to our analysis, MIR155HG could regulate the ICP-related gene CXCL10 by cooperating with the immune gene EBI3 (the COO pattern) (Fig. 6f). We found that the ICP-LncCRCT MIR155HG/CXCL10/EBI3 signature was an independent prognostic factor for overall survival (OS) and could be a key integrated prognostic biomarker in SKCM (Fig. 6g). A lower risk score for MIR155HG/CXCL10/EBI3 was associated with a better prognosis in SKCM patients (*P* = 0.00017; Fig. 6h). The global ICP-LncCRCT MIR155HG/CXCL10/EBI3 could better distinguish the prognosis of SKCM patients compared to single gene (Supplementary Fig. 12). We also found that the ICP-LncCRCT MIR155HG/CXCL10/EBI3 could be an effective prognostic biomarker. The calibration curve showed that the risk score of MIR155HG/CXCL10/EBI3 had a satisfactory fit between the predicted and actual observations. Specifically, the risk score of the ICP-LncCRCT MIR155HG/CXCL10/EBI3 combination could more effectively predict 1-, 3- and 5-year prognoses than could the single ICP or lncRNA alone in SKCM patients (Fig. 6i). Taken together, these results indicated that several ICP-LncCRCTs were associated with survival in all cancer types. MIR155HG/CXCL10/EBI3 could be effective prognostic biomarkers in SKCM. To a certain degree, the whole MIR155HG/CXCL10/EBI3 axis has better predictive performance for survival than single molecules in SKCM.

### Combining several ICP-LncCRCTs with the tumor mutation burden (TMB) improves the prediction of survival in ICI-treated SKCM patients

**Fig. 7:**
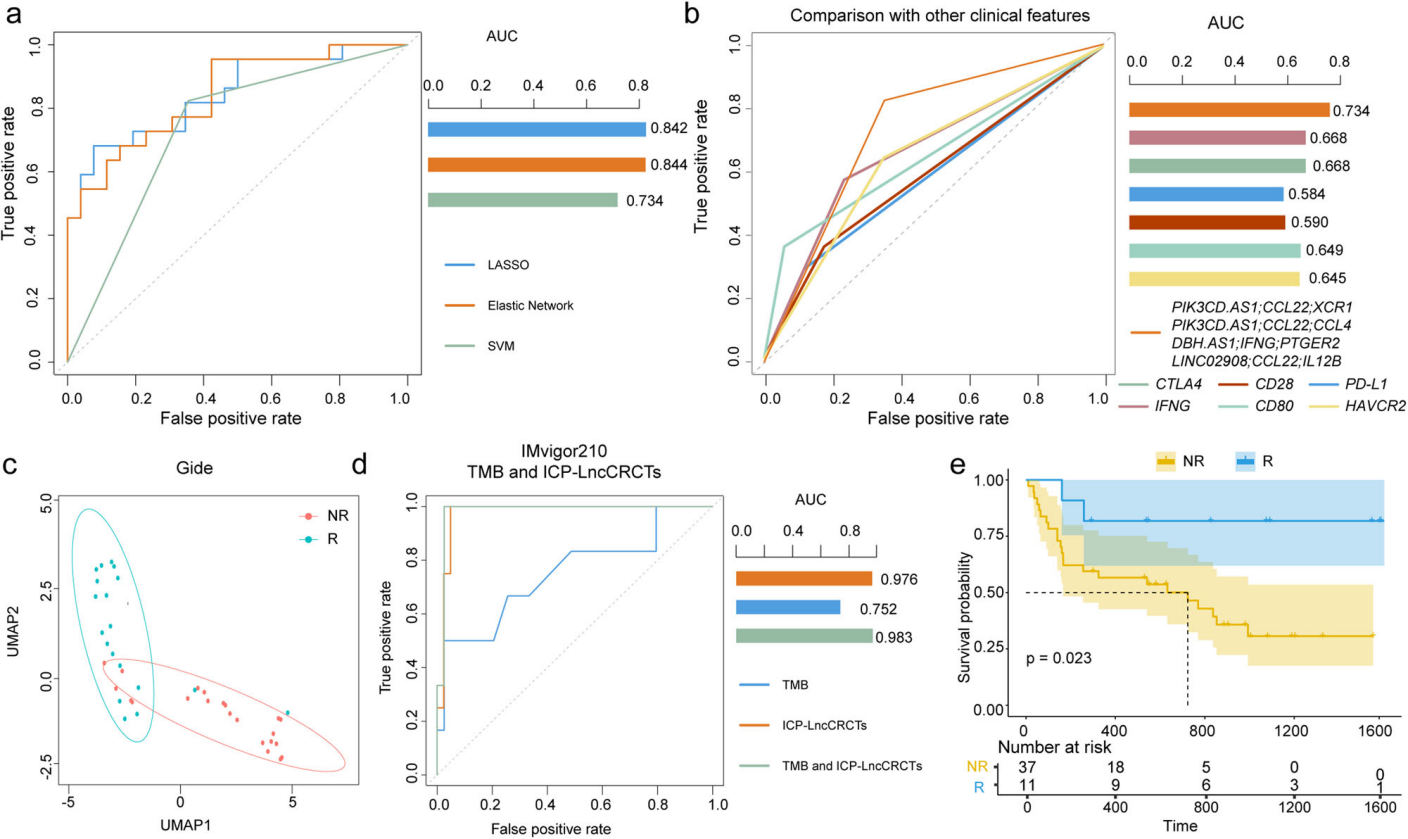
Prediction of ICI response. **a** ROC curves of three machine learning algorithms constructed using ICP-LncCRCTs to predict ICI responses. **b** Comparison of the ability of ICP-LncCRCT to predict ICI response with that of other clinical biomarkers. **c** UMAP dimension reduction and clustering of responders and nonresponders based on the expression of ICP-LncCRCTs in the SKCM dataset (Gide et al. [anti-PD-1]). **d** Impact of the TMB in combination with the ICP-LncCRCT on tumor predictive performance. **e** Kaplan–Meier curves of overall survival in SKCM patients between two groups divided by the expression levels of ICP-LncCRCTs in responders and nonresponders. The survival difference is calculated by log-rank test. Solid blue lines indicate the responding group, and solid yellow lines indicate the nonresponding group. Shaded areas indicate 95% confidence intervals. Asterisks on the curves indicate censoring points.

To evaluate the ability of the ICP-LncCRCTs to predict ICI response, multiple datasets from SKCM were used for analysis. After integrating the five SKCM datasets, only four ICP-LncCRCTs were extracted. Three kinds of machine learning algorithms, LASSO, the elastic network, and SVM, were constructed to predict ICI response. We found that the ROC values were all greater than 0.75 for all three algorithms, especially for the elastic network (AUC = 0.844; Fig. 7a). We also compared the ability of the ICP-LncCRCTs with that of clinical biomarkers to predict ICI response. The area under the curve (AUC) values of the ICP-LncCRCTs were greater than those of several clinical biomarkers, including CTLA4, CD28, PD-L1, IFNG, CD80 and HAVCR2, for predicting ICI response (Fig. 7b). The ICI responders and nonresponders could be well distinguished after UMAP dimension reduction and clustering based on the expression of ICP-LncCRCTs in the SKCM with anti-PD-1 dataset (Fig. 7c). A high TMB is usually considered a clinical biomarker for increased benefit from ICI treatment. However, ICI responders and nonresponders often exhibit significant overlap in TMB, suggesting that the TMB alone is not a sufficient predictor of the ICI response. Therefore, we investigated whether combining ICP-LncCRCTs with TMB-based predictors improved the prediction performance. The AUC improved from 0.752 (only for TBM) and 0.976 (only for ICP-LncCRCTs) to 0.983 when ICP-LncCRCTs were combined with the TMB (Fig. 7d). Additionally, survival was better in the response groups (Fig. 7e). Taken together, our results suggest that (i) several ICP-LncCRCTs could predict ICI response and that (ii) several ICP-LncCRCTs could help improve TMB-based ICI response predictions.

## Discussion

Dysfunction of the ICPs in tumor cells enables them to evade recognition and destruction by immune cells, promoting tumor growth and metastasis^21^. LncRNAs can directly and indirectly regulate ICP genes. Thus, the core regulatory circuitry consisting of lncRNAs, immune genes and ICP genes is crucial for exploring the regulatory mechanisms of ICP molecules in tumors. Herein, a complex computational algorithm was developed to identify and explore the regulatory patterns of ICP-related lncRNAs and ICP-LncCRCTs across cancers (Fig. 1a). Some previous works, such as ImmLnc, had identified immune-related lncRNAs^22^. Our research, however, has a deeper approach to understanding the role of lncRNAs in ICI treatment by concentrating on ICP-related lncRNAs. We also considered our work could more accurately focused extract ICP-related lncRNAs from a large number of immune-related lncRNAs (Supplementary Fig. 13). In addition, we not only identified immune-related lncRNAs but also, more importantly, identified regulatory associations and patterns among lncRNAs, immune-related genes, and ICP genes. We have further investigated the causal association between ICP and lncRNAs. The ability of the ICP-LncCRCT signature to predict patient prognosis and immunotherapy response was also validated in SKCM. Our results demonstrated a complex relationship between ICP, lncRNAs, and immune genes and may help to evaluate ICI response in cancer patients.

To improve our understanding of the impacts of lncRNAs on ICP-related gene activity mediated by immune genes and their role in immune regulation in cancer, we identified ICP-related lncRNAs and constructed ICP-related lncRNA-mediated core regulatory circuitry triplets (ICP-LncCRCTs). The page-rank algorithm was used to extract ICP-proximal genes and lncRNAs. Although the page-rank algorithm is a network propagation algorithm that is based on degree, we also found that the betweenness (Betweenness of a node is a measure of the extent to which a node acts as an intermediary in a network, measuring the importance and control a node has in connecting other nodes to each other in the network) of the optimizing genes was greater than that of the other genes in all cancer types (*P* < 0.001, K-W test, Supplementary Fig. 14). The top 200 immunity genes and lncRNAs with high betweenness in the co-expression network were identified in our work. Next, we proposed potential regulatory patterns dominated by ICP-related lncRNAs and inferred the probable regulatory pattern for each ICP-LncCRC based on the Bayesian network and maximum likelihood estimation. The COO and LIG regulatory patterns had the largest and the smallest proportions, respectively, across cancers. In our analysis, we considered only four basic and major assumed regulatory patterns. There is no denying that other possibilities exist for lncRNAs to regulate ICP regulatory patterns. Although there are certain limitations associated with these assumed regulatory patterns, such as Bayesian network inference processing, which also relies on prior knowledge, these patterns could still provide perspectives for exploring immune regulation and ICI response. These causal associations should be further validated in vitro or in vivo.

Making effective biomarkers useful for predicting patient prognosis and ICI response has always been a major challenge in precision medicine using immunotherapy. Although several biomarkers, such as PD-L1, TMB, and some genes, have been approved by the Food and Drug Administration (FDA) or through experiments, these biomarkers lack universality and specificity. Thus, identifying more accurate biomarkers for predicting ICI response is urgently needed. According to our analysis, compared with a single gene, ICP-LncCRCTs could better predict one-, three- and five-year OS in SKCM patients. In the context of the five integrated SKCM datasets, ICP-LncCRCTs exhibited superior predictive power compared to several clinical biomarkers, as assessed by various machine learning algorithms. Specifically, ICP-LncCRCTs can help improve TMB-based ICI response predictions. In future work, the ability of several ICP-LncCRCTs to predict OS should be validated in additional cancer types following additional data generation and development with respect to ICI response.

In our work, we used partial correlation to exclude the influence of tumor purity in calculating the associations between ICP and lncRNA. The result indicated that there is no significant difference in the partial correlation coefficients between high- and low-tumor purity groups in half of the cancer types (Supplementary Fig. 15). More attempts, validations, and methods were also needed to further eliminate the influence of tumor purity. In addition, more silico algorithms and histology slides should be used to validate the estimation of tissue content, such as tumor purity and immune cell purity. Although bulk RNA-seq data were used in the present study, the results could be validated in immune cell lines and scRNA-seq datasets. With the continuous increase in the amount of scRNA-seq data with additional samples, cancer types, and clinical information on immune cells, more accurate information on the regulatory associations between ICP and lncRNAs could be obtained and analyzed.

In summary, we further revealed the associations among ICP, lncRNAs, and immune genes in cancers by integrating bulk, single-cell, and immune cell line datasets. The ICP-LncCRCT CXCL10-MIR155HG-EBI3 showed a more accurate predictive ability for one-, three-, and five-year prognoses in melanoma patients than single-molecule therapy. We also validated that several ICP-LncCRCTs could effectively predict ICI response. Specifically, combining ICP-LncCRCTs with the tumor mutation burden (TMB) improved the ability of ICI-treated melanoma patients to predict ICI efficacy. These findings could lead to the identification of effective candidates for further exploration of immune function and regulation of lncRNAs and could prove valuable in future immunotherapeutic strategies.

## Methods

### Data collection

The gene expression profiles for 18 cancer types from The Cancer Genome Atlas (TCGA) portal and noncancerous tissues from the Genotype Tissue Expression (GTEx) consortium were downloaded from the UCSC Xena platform (http://xena.ucsc.edu/). We also downloaded raw RNA-seq data from several independent datasets obtained from other public sources to validate the algorithm. The gene expression profiles of GBM and PRAD from the International Cancer Genome Consortium (ICGC) were used as independent datasets to validate the results. Batch effects were removed between multiple independent datasets. Specifically, batch effects were removed from the GTEx samples compared with the normal samples in the TCGA. Normalized expression profiles were subjected to log2 transformation. For the gene expression profiles, we removed the genes that were not expressed in more than 70% of the samples. The details of each dataset we used are described in Supplementary Tables 1 and 2. Clinical data were also downloaded from the UCSC Xena platform. To identify ICP genes, we searched PubMed using a list of keywords, such as ‘immune checkpoint’, ‘immunotherapy’, and ‘ICP’. Additionally, we collected information via handbooks or website instructions from multiple companies. We extracted experimentally supported ICPs “by hand,” that is, by manually curating them from published papers. All the selected studies were reviewed by at least two researchers. We extracted experimentally supported ICPs, which were confirmed by strong experimental evidence, including RNA interference, in vitro knockdown, western blot, real-time quantitative polymerase chain reaction (qRT–PCR), and luciferase reporter assays.

### Differential expression analysis of lncRNAs, immune genes, and ICP genes in different cancers

We identified differentially expressed lncRNAs, immune genes, and ICP genes between cancer and normal tissue samples across different types using the limma package in R software. To mitigate the influence of varying sample sizes for each cancer type, we utilized the *p* value distribution, which was generated from 1000 permutations of each cancer, to determine the final cutoff. Initially, the sample labels were randomly perturbed, and the differential expression analysis was re-performed to calculate the randomized *p* value. If the randomized *p* value was smaller than the original *p* value, it was recorded once. This perturbation was repeated 1000 times, and the final adjusted *p* value for each gene was determined by the proportion of 1000 random perturbations, which yielded a *p* value lower than the original one. Multiple tests were performed to correct the *p* values. LncRNAs, immune genes, and ICP genes with false discovery rate (FDR) values less than 0.01 were identified for further analysis.

### Identification of lncRNAs, immune genes, and ICP genes that may have ternary regulatory associations

First, we constructed a co-expression network of lncRNAs, immune genes, and ICP genes based on Pearson correlation coefficients between genes, which were used as the weights of the network edges. Mutual information (MI) was used to validate the correlations among lncRNAs, immune genes, and the ICP gene. To reduce the influence of sample size for each cancer type, the *p* value distribution generated from 1000 permutations of each cancer was used to determine the final cutoff. We identified lncRNAs and immunity genes that were closely associated with the ICP genes via network propagation using the page-rank algorithm from the NetworkX Python module. We used one for the ICP genes and zero for all the other genes in the network as inputs for the personalization parameter in the page-rank algorithm. Default settings were used for any other parameters for the page-rank algorithm. After network propagation, we considered the top 200 immunity genes or lncRNAs with the highest influence scores as genes closely related to the ICP genes.

Next, we used partial correlation analysis to exclude the influence of tumor purity and further screened the correlations between the three types of genes. The expression of the lncRNA, immune gene, and ICP gene was defined as L(i) = (l1, l2, l3, …, lm), G(i) = (g1, g2, g3, …, gm), and C(i) = (c1, c2, c3, …, cm), respectively. The tumor purity scores across n patients were defined as *P* = (p1, p2, p3, …, pn). We first calculated the partial correlation coefficient between the expression of lncRNA i and the expression of the ICP gene j by considering tumor purity as a covariable:

$${{{\rm{PCC}}}}({\it{ij}})=\frac{{{{{\rm{R}}}}}_{{{{\rm{LC}}}}}-{{{{\rm{R}}}}}_{{{{\rm{LP}}}}}* {{{{\rm{R}}}}}_{{{{\rm{CP}}}}}}{\sqrt{1-{{{{\rm{R}}}}}_{{{{\rm{LP}}}}}^{2}}* \sqrt{1-{{{{\rm{R}}}}}_{{{{\rm{CP}}}}}^{2}}}$$

where RLC, RLP, and RCP are the correlation coefficients between the expression of lncRNA *i* and the ICP gene *j*, the expression of lncRNA *i* and tumor purity, and the expression of the ICP gene j and tumor purity, respectively.

Then, immune genes regulated by lncRNAs and ICP genes were screened using GSEA enrichment analysis to obtain immune genes that could be enriched in 18 immune pathways.

Finally, *S* scores were assigned to the gene pairs composed of lncRNAs and ICP genes based on the results of GSEA and partial correlation analysis.

$${\it{S}}=\mathop{\sum }\limits_{{\it{i}}=1}^{{\it{n}}}{{{{\rm{w}}}}}_{{\it{i}}}(-\log 10\left({{{{\rm{P}}}}}_{1{{{\rm{i}}}}}\right)* {{{\rm{sign}}}}\left({{{{\rm{cor}}}}}_{1{{{\rm{i}}}}}\right)-\log 10\left({{{{\rm{P}}}}}_{2{{{\rm{i}}}}}\right)* {{{\rm{sign}}}}({{{{\rm{cor}}}}}_{2{{{\rm{i}}}}}))$$

where *n* is the number of immunity genes regulated by the gene pairs composed of lncRNAs and ICP genes, wi is the enrichment score of immunity genes in the GSEA enrichment results, P1i is the partial correlation *p* value of lncRNAs and immunity genes, P2i is the partial correlation *p* value of ICP genes and immunity genes, cor1i is the partial correlation coefficient of lncRNAs and immunity genes, and cor2i is the partial correlation coefficient of ICP genes and immunity genes.

After scoring each gene pair composed of the lncRNA and ICP genes, 1000 random perturbations were performed by perturbing the sample labels, and the FDR was calculated by Benjamini and Hochberg (BH) correction to obtain the gene pairs composed of lncRNAs and ICP genes with FDR < 0.01. By partial correlation analysis, GSEA, S score, and random perturbation, we obtained lncRNAs, immunity genes, and ICP genes with possible ternary regulatory associations. We named these regulatory associations among lncRNA, immunity gene, and ICP gene as ICP-related lncRNA-mediated core regulatory circuitry triplets (ICP-LncCRCTs).

### Construction of a possible regulatory model among lncRNAs, immunity genes, and ICP genes in ICP-LncCRCTs

Based on the previously identified lncRNAs, immunity genes, and ICP genes with possible ternary regulatory associations, we constructed ICP-related lncRNA-mediated core regulatory circuitry triplets (ICP-LncCRCTs) and proposed four possible regulatory models: INDEP, LGI, LIG and COO regulatory patterns. For each of the proposed patterns, we defined the joint probability distribution based on the assumption of standard Markovian characterization as follows:

$${P}_{{INDEP}}(L,C,M)=P(L)P(C{{{\rm{|}}}}L)P(M{{{\rm{|}}}}L),$$

$${P}_{{COO}}(L,C,M)=P(L)P(M)P(C{{{\rm{|}}}}L,M),$$

$${P}_{{LGI}}(L,C,M)=P(L)P(M{{{\rm{|}}}}L)P(C{{{\rm{|}}}}M),$$

$${P}_{{LIG}}(L,C,M)=P(L)P(C{{{\rm{|}}}}L)P(M{{{\rm{|}}}}C),$$

where *L* represents the expression value of the lncRNA, C represents the expression value of the ICP, and M represents the expression value of the immune genes. We assigned each ICP-LncCRCT to the corresponding pattern by the MLE method. Subsequently, we calculated the Akaike information criterion (AIC) score and Akaike weights (w(AIC)).

$${{AIC}}_{j}=-2\log {L}_{j}+2{K}_{j}$$

$${w}_{j}({AIC})=\frac{{e}^{-\frac{1}{2}{\Delta }_{j}({AIC})}}{{\sum }_{j=1}^{4}{e}^{-\frac{1}{2}{\Delta }_{j}({AIC})}},{w}_{j}({AIC})\in (0,1)$$

$${\Delta }_{j}({AIC})={{AIC}}_{j}-\min ({AIC})$$

where *L*_*j*_ is the maximum likelihood for pattern *j*. Finally, the pattern with the lowest AIC and largest w(AIC) was selected as the best pattern for each ICP-LncCRCT. The pattern selection process was performed using the R package bnlearn.

### Recognition of pattern-variable gene pairs composed of lncRNAs and ICP genes

Immunity genes produce a marked effect on the process through which lncRNAs regulate ICPs. In various cancers, the same lncRNA-ICP gene pair can form ICP-LncCRCTs with differing patterns, shaped by the diverse immune genes involved. We defined lncRNA-ICP gene pairs that occurred in more than or equal to five cancers and had different patterns of ICP-LncCRCT due to different immune genes as pattern-variable lncRNA-ICP relationship pairs.

### Correlations between ICP-related lncRNAs and antitumor immune activity in cancers

We evaluated the immunological characteristics of the ICP-related lncRNAs by calculating correlations with the following factors: (i) Immune cell infiltration score from TIMER. (ii) Antitumor immunoreactivity was measured by the MHC score and CYT score according to the mean expression of the corresponding markers^23,24^. (iii) Expression of immune cell markers. (iv) Expression in immune cell lines. (v) IHC and IF staining were performed with a Human Protein Atlas (https://www.proteinatlas.org/). Spearman rank correlations were calculated between the expression of each lncRNA and the TIMER score, MHC score, CYT score, and expression of immune cell markers. Kruskal‒Wallis (K-W) tests were performed to evaluate the differences in the expression of each lncRNA in 19 immune cell lines^25^.

### Obtaining and analyzing the scRNA-seq data

The scRNA-seq expression profile data of seven cancer types and 10 datasets were downloaded from the Gene Expression Omnibus (Supplementary Table 3, GEO, https://www.ncbi.nlm.nih.gov/geo). The preprocessed gene expression matrix and cell annotation information were encapsulated using the R package Seurat^26^. The marker genes of specific cell types collected from published literature^27,28^ were used to define cell clusters. The ‘GSVA’ package was used for single sample gene set enrichment analysis (ssGSEA) to evaluate the gene set enrichment score of each cell. The calculated ssGSEA scores are displayed in the UMAP graph. The difference in lncRNA expression among cell types was tested by the K-W test. A common computational pipeline was also used to identify ICP-LncCRCTs across cancer types utilizing scRNA-seq data to analyze various cell types.

### Survival analysis of patients with important ICP-LncCRCTs in multiple cancer types

The association between the expression of ICP-related lncRNAs in ICP-LncCRCTs and survival was assessed using univariate Cox regression analysis and the log-rank test. Candidate prognostic ICP-related lncRNAs were identified by *P* < 0.05 for univariate Cox regression analysis and *P* < 0.05 for the log-rank test.

For ICP-LncCRCTs, we assigned risk scores to each patient based on linear combinations of the expression of each gene in the ICP-LncCRCTs weighted by the regression coefficients from the multivariate Cox regression analyses. The risk scores for each sample were defined as follows:

$${{{\rm{Risk}}}}=\mathop{\sum }\limits_{{\it{j}}=1}^{3}{{{{\rm{\beta }}}}}_{{\it{j}}}* {\exp }_{{\it{j}}}$$

where *n* is the number of genes in each ICP-LncCRCT, *β*_*j*_ is the regression coefficient for the multivariate Cox regression analysis, and exp_*j*_ is the expression of the gene_*j*_. Patients were divided into high- and low-risk groups based on the median risk score, and differences in survival were analyzed using the log-rank test. The calibration curves for the probability of OS showed that the nomogram’s prediction matched the actual observation^29^.

### Prediction of ICI response in SKCM based on ICP-LncCRCT

Five datasets of SKCM treated with immunotherapy were obtained, and batch effects were removed using the R package “sva” (Supplementary Table 4). Seventy percent of the samples were used as the training set, and the remaining samples were used as the test set. After data processing, the expression of lncRNAs, immune genes, and the ICP gene in 216 prognosis-related ICP-LncCRCTs was found in all six datasets.

The expression of four ICP-LncCRCTs, three lncRNAs, three immunity genes, and two ICP genes, was extracted from all the datasets via LASSO regression analysis. We further tested the predictive performance of the models based on the expression of four prognosis-related ICP-LncCRCTs using LASSO regression analysis, elastic network analysis, and support vector machine (SVM) analysis. The receiver operating characteristic (ROC) curve was used to evaluate the predictive ability of the model using the R package “ROCR”. We compared the predictive performance of models based on ICP-LncCRCTs and other clinical indicators (PD-L1, IFNG, CD28, CD80, HAVCR2, and CTLA4) using SVM models. We also analyzed the ability of the IMvigor210 dataset to predict ICI responses using TMB and the mean expression level of ICP-LncCRCTs. For the IMvigor210 dataset, the mutation burden per megabase was used as the TMB.

### Statistics and reproducibility

Statistical analyses were conducted using R software (version 4.3.1). We use the limma package to screen for differentially expressed genes. For the correlation between genes, we use the cor.test function to calculate the Pearson correlation coefficient. The difference between lncRNA and known lncRNA is analyzed by Fisher’s test, and Fisher’s test is performed by the fisher.test function. The significance of the intersection of sets is analyzed by the hypergeometric test, and the hypergeometric test is performed by the phyper function. The difference in lncRNA expression and ssGSEA score among multiple types of cells is analyzed by the Kruskal–Wallis test, and the Kruskal–Wallis test is performed by the Kruskal test function. The comparison of survival curves in survival analysis is analyzed by the log-rank rank test, and the log-rank rank test is performed by the ggsurvplot function in the survminer package.

### Ethics approval and consent to participate

All relevant ethical regulations were followed in the original study of the datasets, and the authors of the source studies also obtained informed consent from participants.

### Reporting summary

Further information on research design is available in the Nature Portfolio Reporting Summary linked to this article.

### Supplementary information

Supplementary Information

Description of Additional Supplementary Files

Supplementary Data 1

Supplementary Data 2

Reporting Summary

## Data Availability

The TCGA datasets were downloaded from the UCSC Xena platform (http://xena.ucsc.edu/). scRNA-seq data^28,30–37^ was obtained from GEO with the accession number GSE127471, GSE117570, GSE69405, GSE75688, GSE118389, GSE125449, GSE81861, GSE103322 and GSE72056. Immunotherapy response data^38–40^ was obtained from Tumor-Immune Dysfunction and Exclusion (http://tide.dfci.harvard.edu/). The analysis results associated with this paper are available on GitHub (https://github.com/GaoYueWorkspace/ICP-related-lncRNAs/Bayes network ICP-related lncRNA/Data) and are publicly accessioned via Zenodo^41^ (10.5281/zenodo.10726010). The numerical source data for the graphs in the main and Supplementary Figs. can be found in Supplementary Data 1, 2.

## Code availability

The R code used in the analysis of the data is available on GitHub (https://github.com/GaoYueWorkspace/ICP-related-lncRNAs/Bayes network ICP-related lncRNA/Code) and is publicly accessioned via Zenodo^41^ (10.5281/zenodo.10726010).
