# Supplementary material for: The landscape of immune checkpoint-related long non-coding RNAs core regulatory circuitry reveals implications for immunoregulation and immunotherapy responses
Source: Commun Biol. 2024 Mar 14;7:327. doi: 10.1038/s42003-024-06004-z (PMC10940638; doi:10.1038/s42003-024-06004-z)
Supplement: Supplementary file 1 — Supplementary Information [file 42003_2024_6004_MOESM1_ESM.pdf]

Supplementary Figure 1

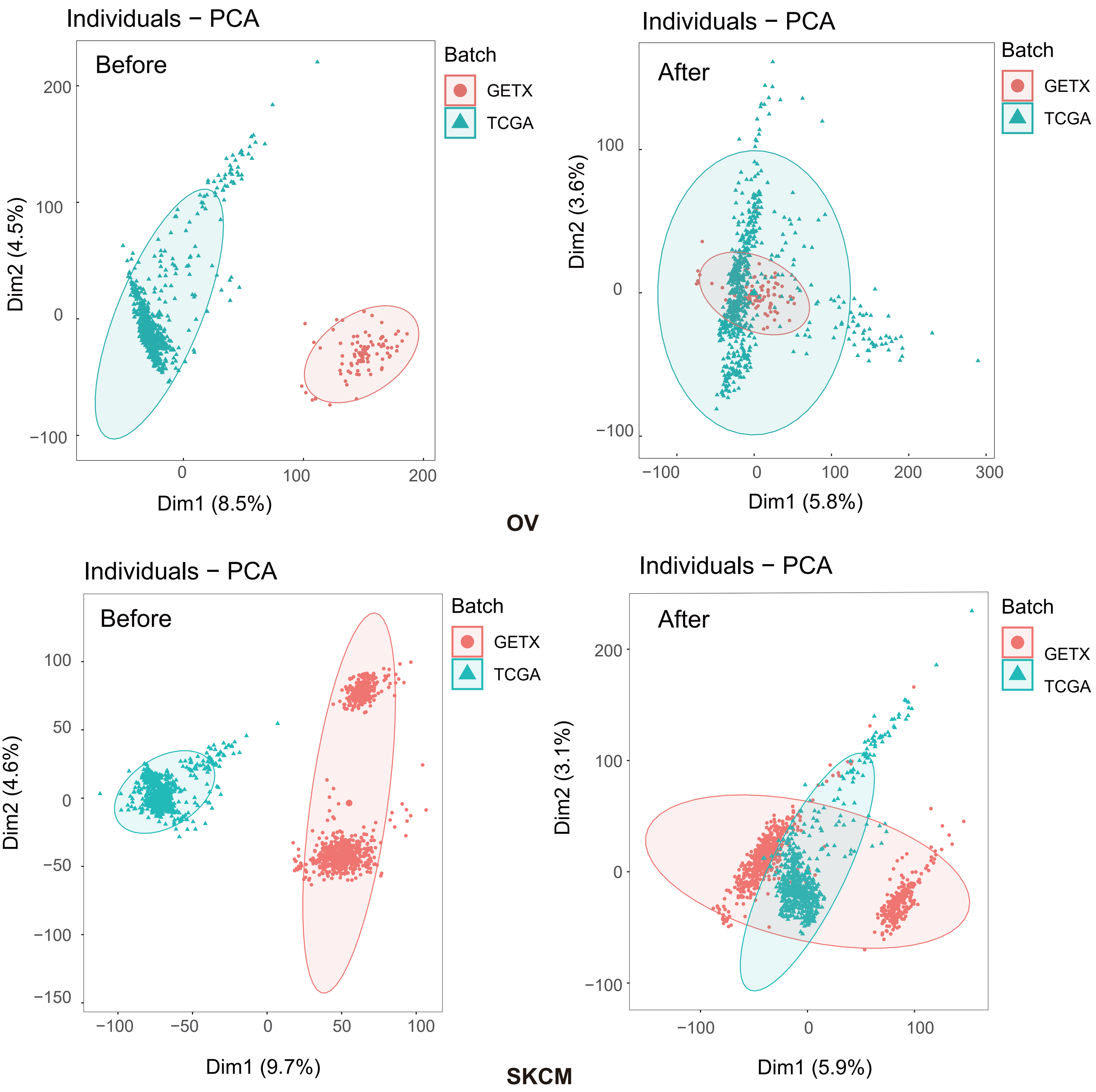

Supplementary Figure 1. Two cancers, OV and SKCM, are combined with GTEx for batch removal.

Supplementary Figure 2

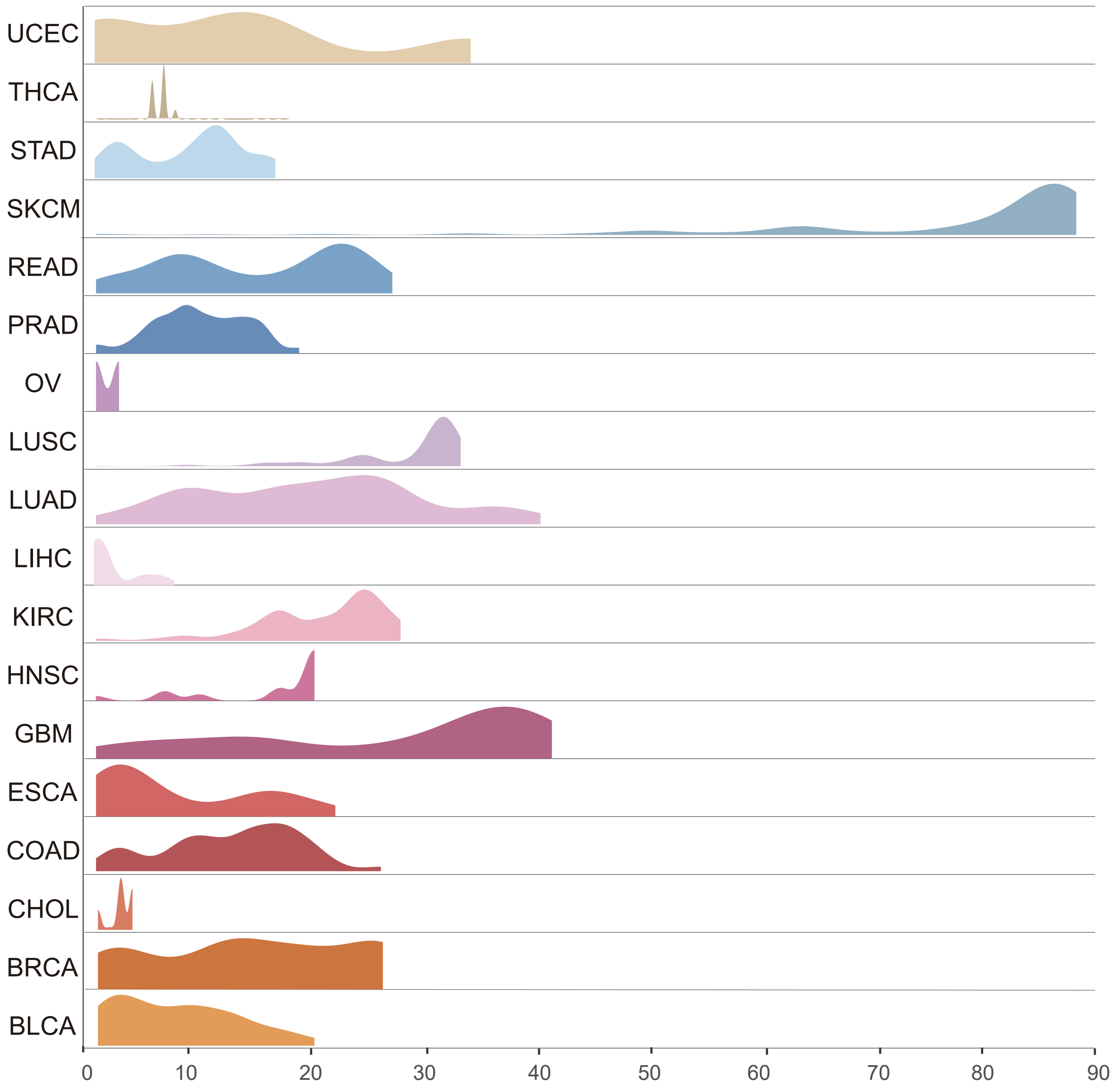

Supplementary Figure 2. Distribution of immune genes in ICP-LncCRCTs across all cancers, with different colors representing different cancers.

Supplementary Figure 3

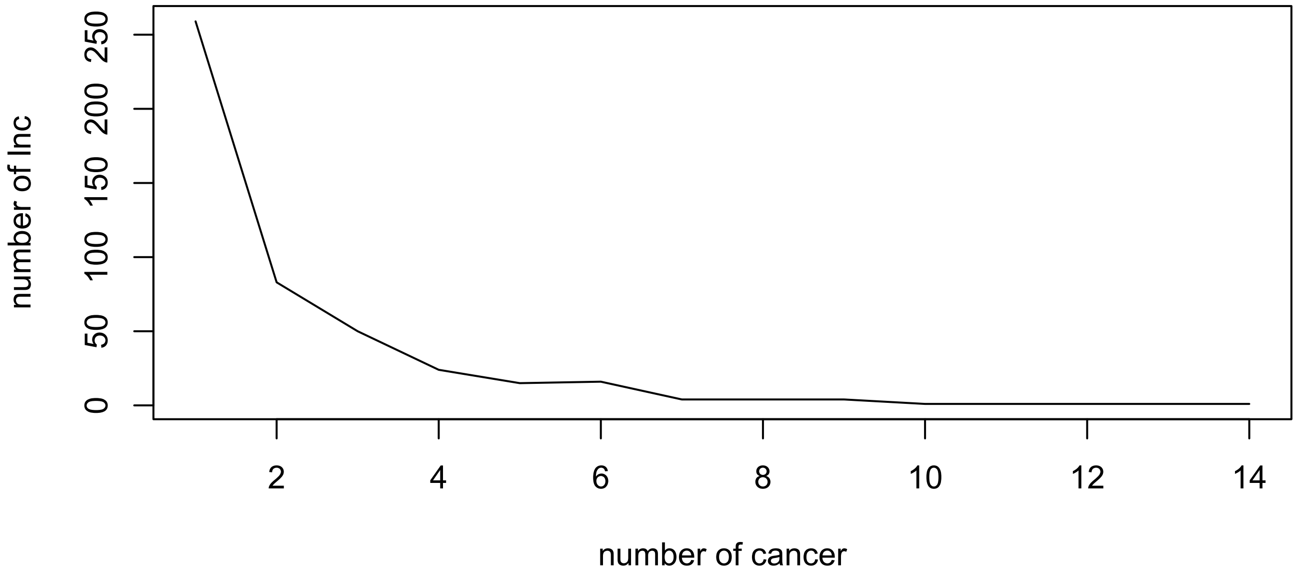

Supplementary Figure 3. The numbers of common lncRNAs are similar when five to 6~14 are changed.

Supplementary Figure 4

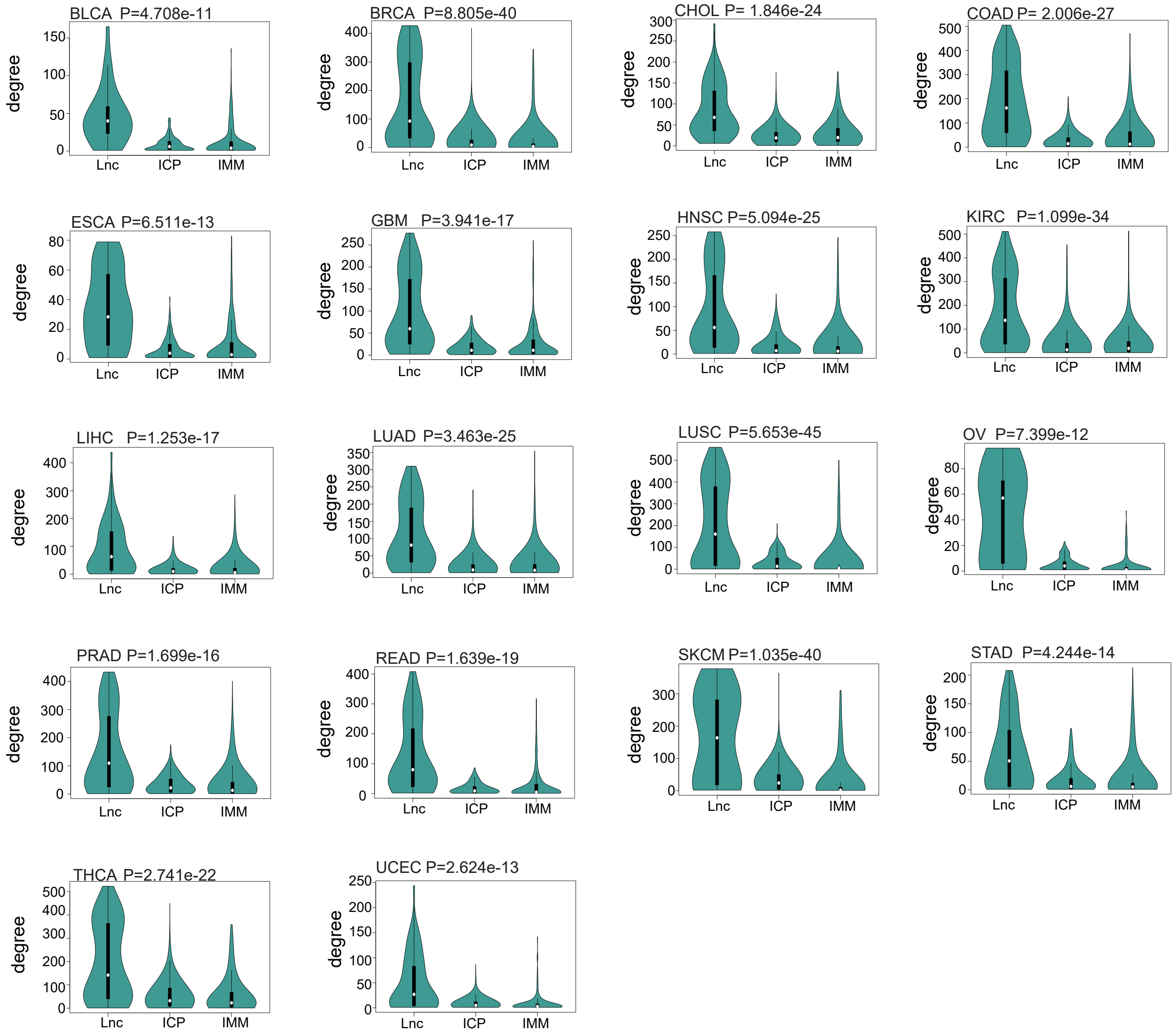

Supplementary Figure 4. Degree distributions of lncRNA, ICP and IMM in the ICP-LncCRCTs network. P-values for the K-W test are at the upper left. Black boxes indicate the interquartile range of the data. White dots indicate the median. Black vertical lines indicate 95% confidence intervals. The width of the violin plot indicates the density of the data.

Supplementary Figure 5

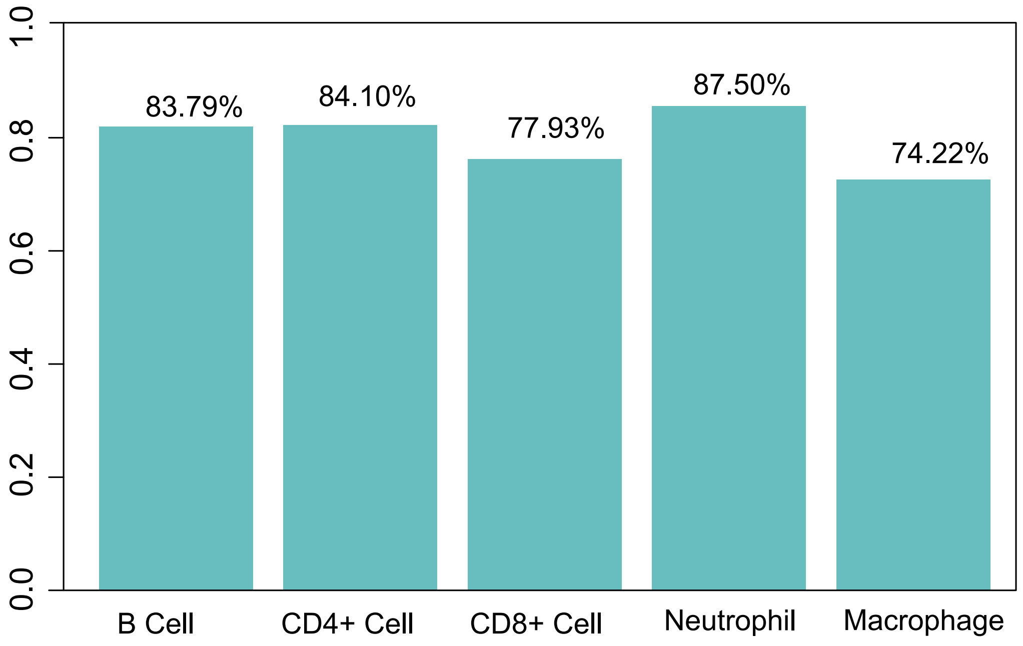

Supplementary Figure 5. The vast majority of ICP-related lncRNAs are associated with immune infiltration. The bar graph demonstrates the proportion of ICP-related lncRNAs associated with infiltration to all ICP-related lncRNAs in the five immune cell types.

Supplementary Figure 6

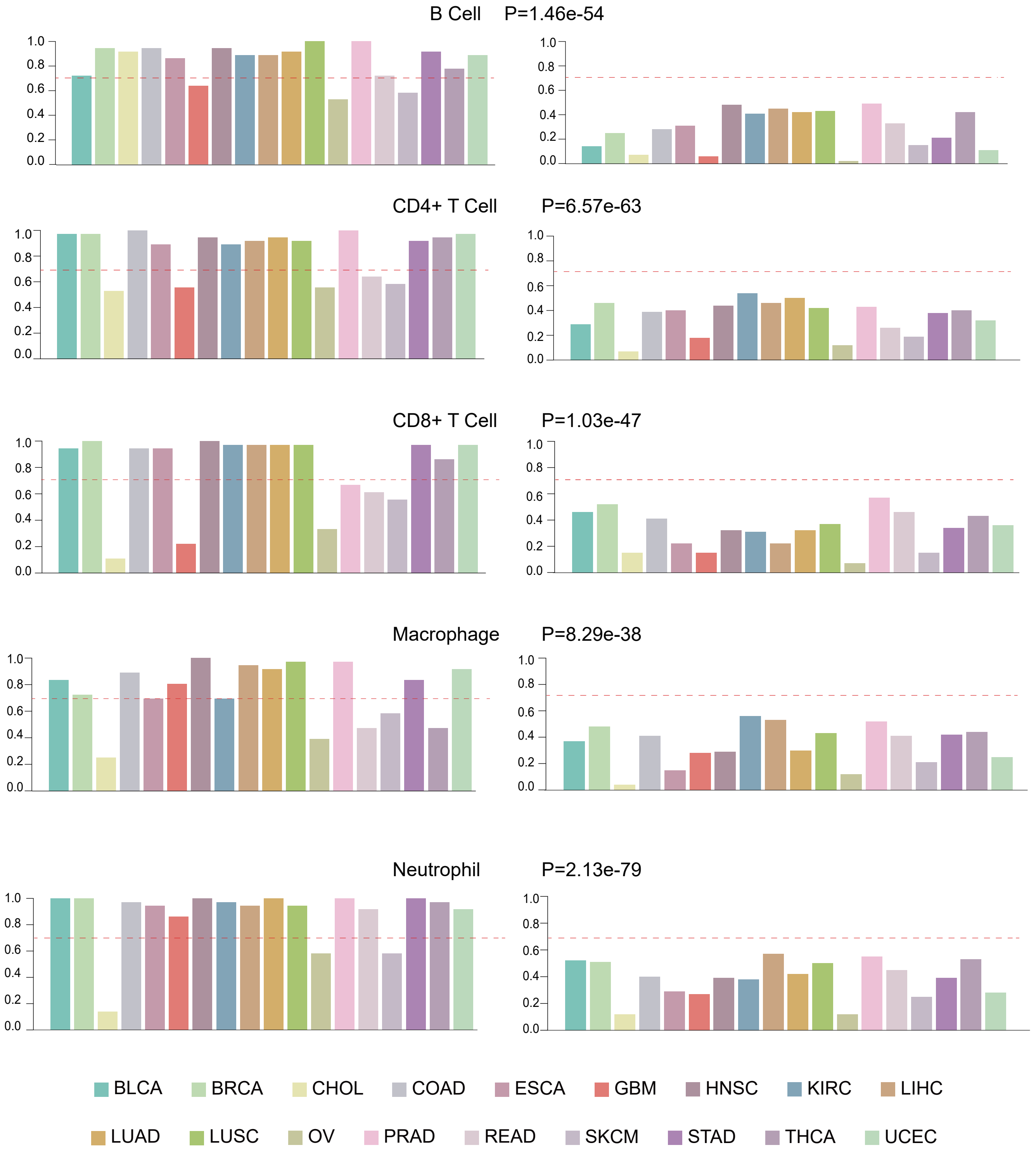

Supplementary Figure 6. Comparison of immune infiltration correlation of common ICP-related lncRNAs with other lncRNAs. Different colors represent different types of cancer.

Supplementary Figure 7

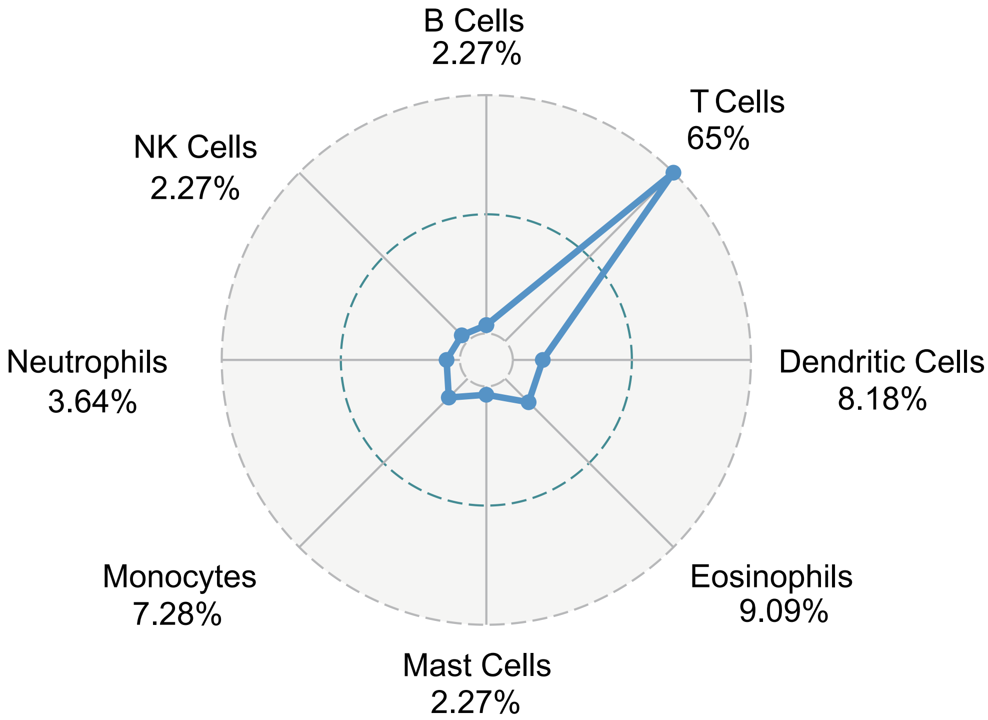

Supplementary Figure 7. Radar plots indicate the proportion of highly expressed ICP-related lncRNAs in different cells.

Supplementary Figure 8

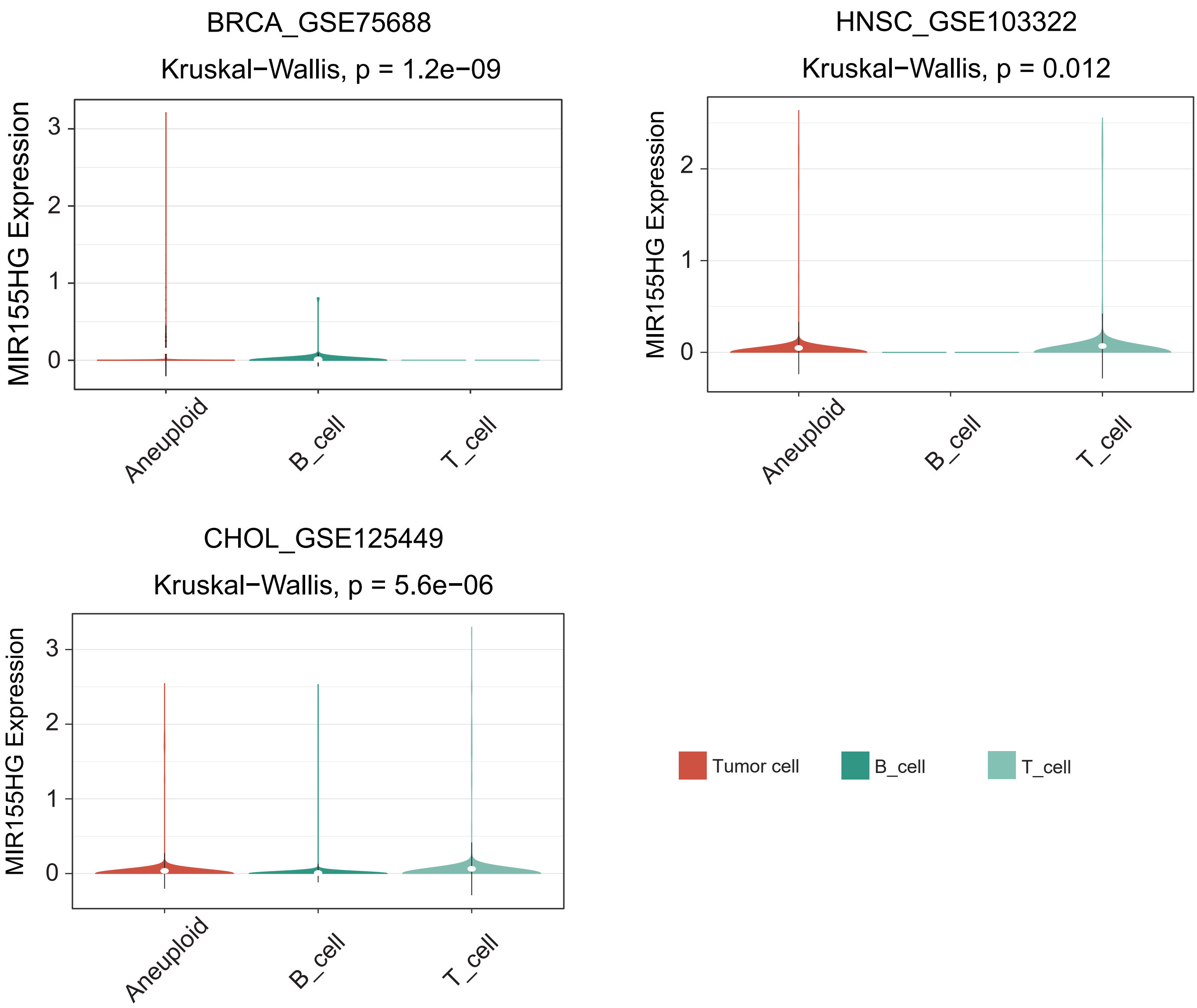

Supplementary Figure 8. Violins indicate the differential expression of MIR155HG in different types of immune cells in the GSE75688, GSE125449 and GSE103322 datasets. White dots indicate the median. Black vertical lines indicate 95% confidence intervals. The width of the violin plot indicates the density of the data.

Supplementary Figure 9

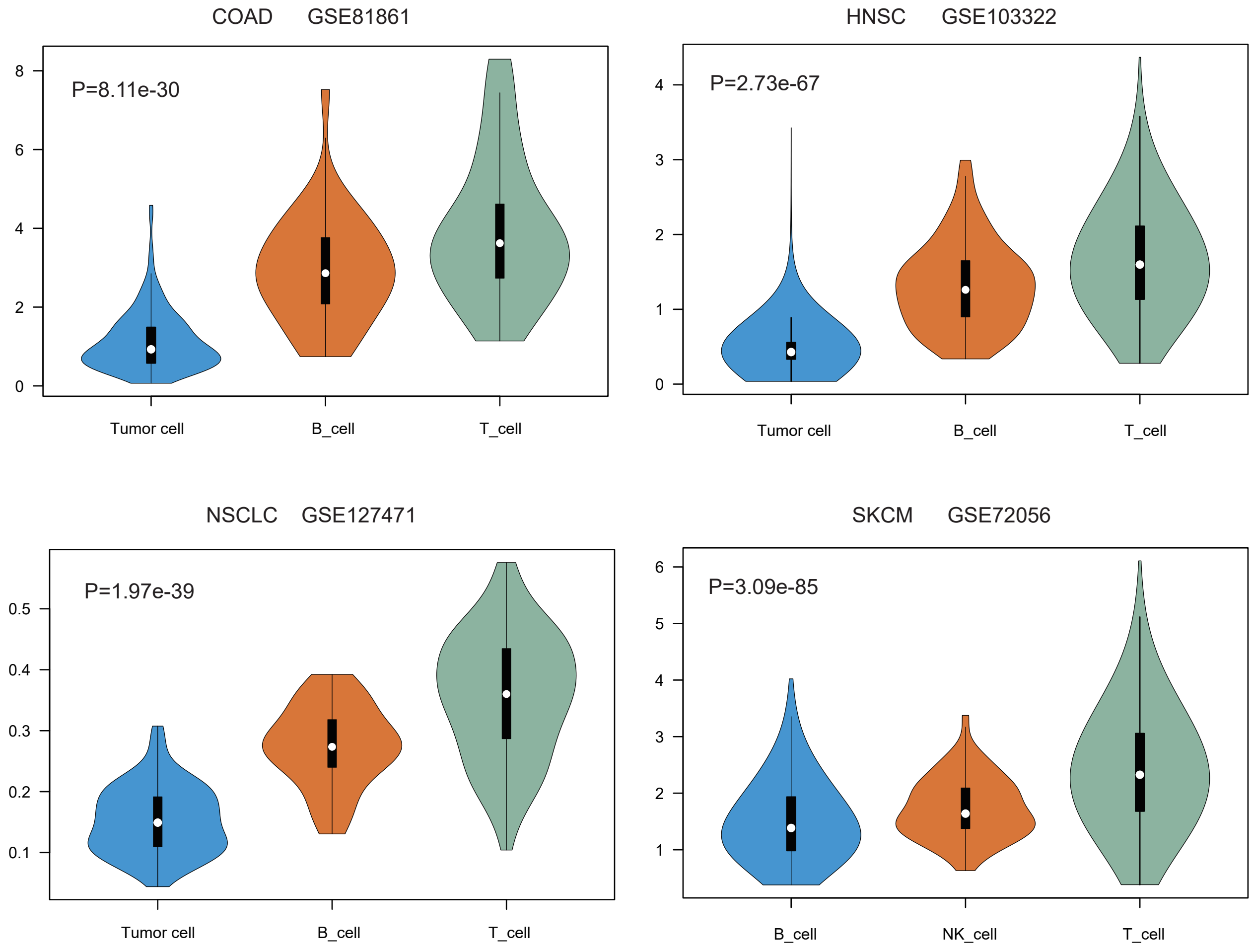

Supplementary Figure 9. Violins indicate the differential expression of lncRNA-ICP relationship pairs between different cell types in different single-cell datasets. Black boxes indicate the interquartile range of the data. White dots indicate the median. Black vertical lines indicate 95% confidence intervals. The width of the violin plot indicates the density of the data.

Supplementary Figure 10

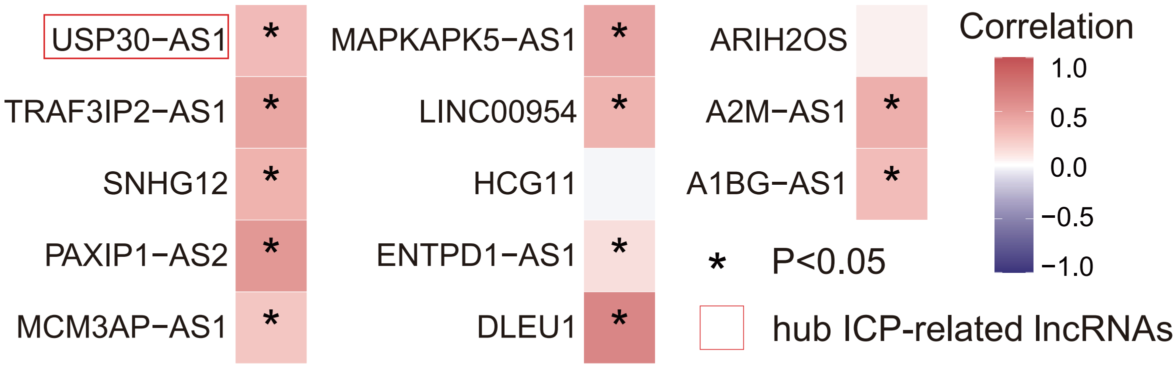

Supplementary Figure 10. Common ICP-related lncRNAs clustered with MIR155HG show a close co-expression. Correlation is assessed using the Pearson correlation coefficient.

Supplementary Figure 11

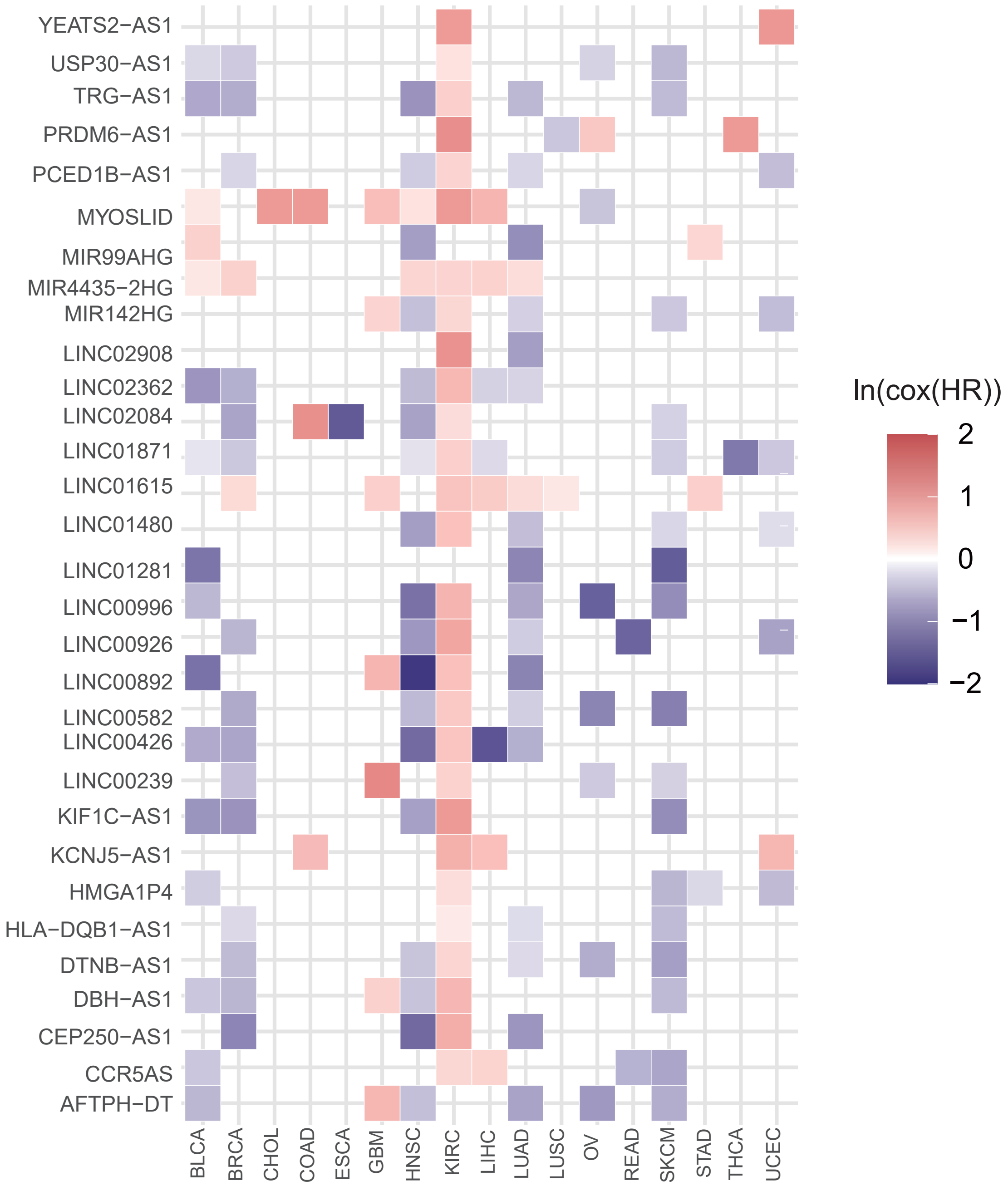

Supplementary Figure 11. Prognosis-related ICP-related lncRNAs. Shades of color indicate the strength of the correlation with prognosis.

Supplementary Figure 12

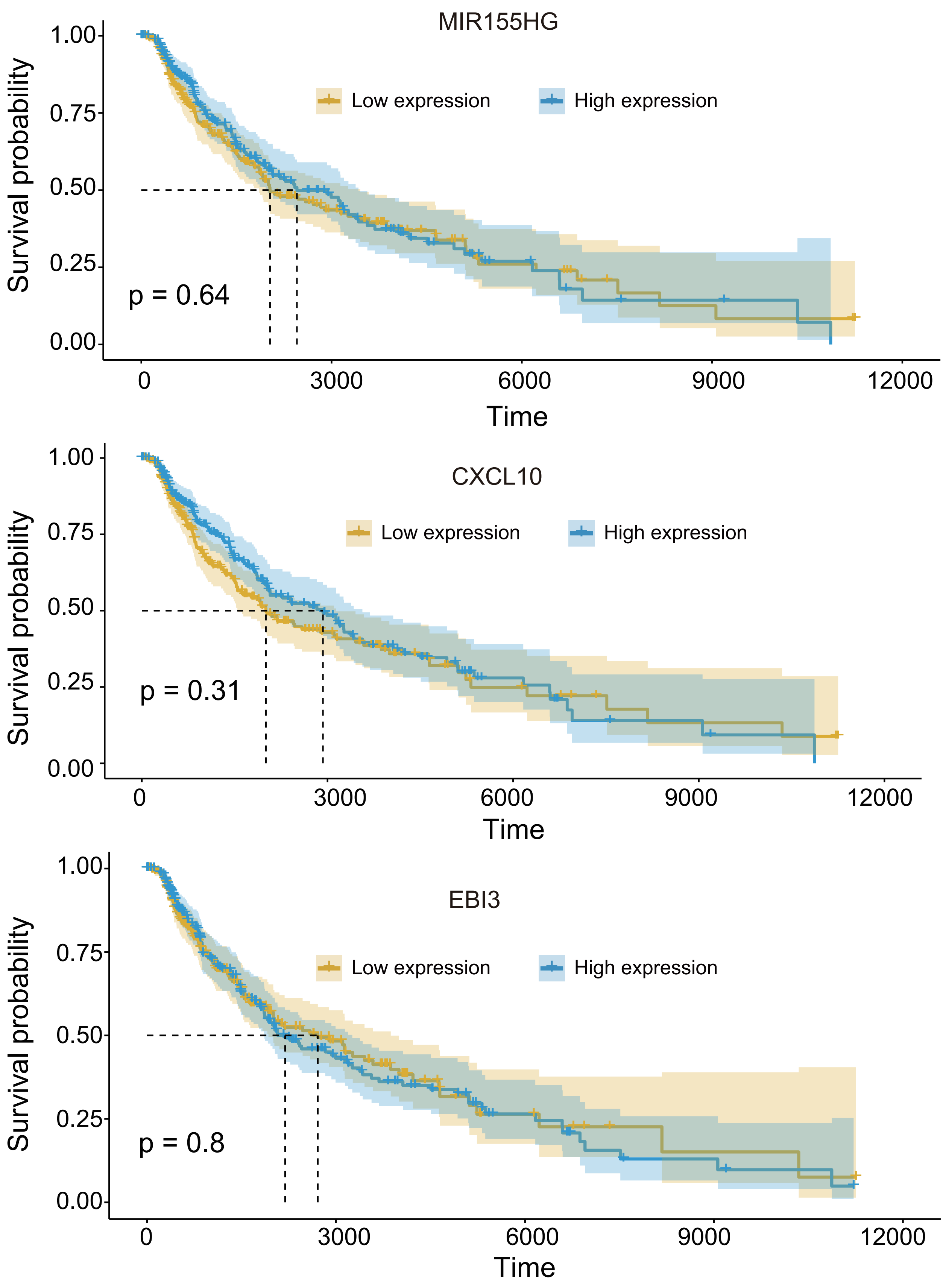

Supplementary Figure 12. KM curves of each gene in ICP-LncCRCT MIR155HG/CXCL10/EBI3. Solid blue lines indicate the high expression group and solid yellow lines indicate the low expression group. Shaded areas indicate 95% confidence intervals. Asterisks on the curves indicate censoring points.

Supplementary Figure 13

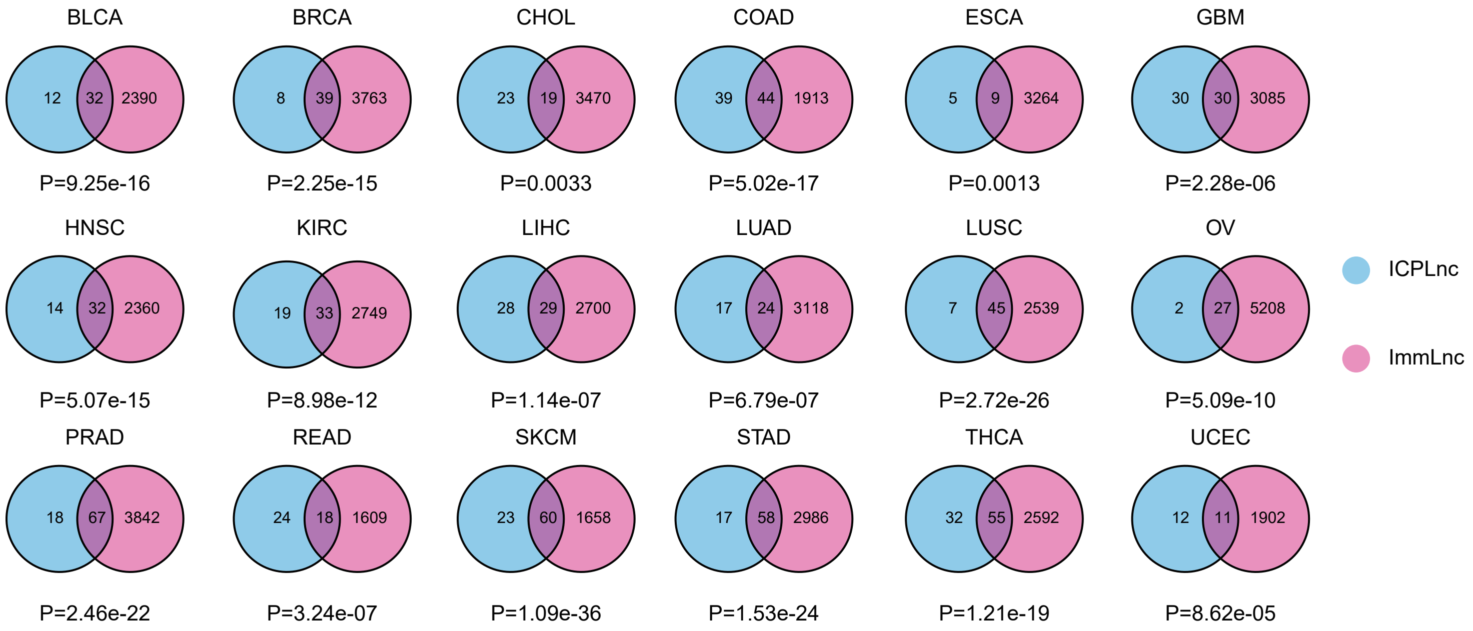

Supplementary Figure 13. Comparison of ICP-associated lnc with immune-associated lnc in ImmLnc work. P-values are calculated using the hypergeometric test with a background gene cunt of 13953.

Supplementary Figure 14

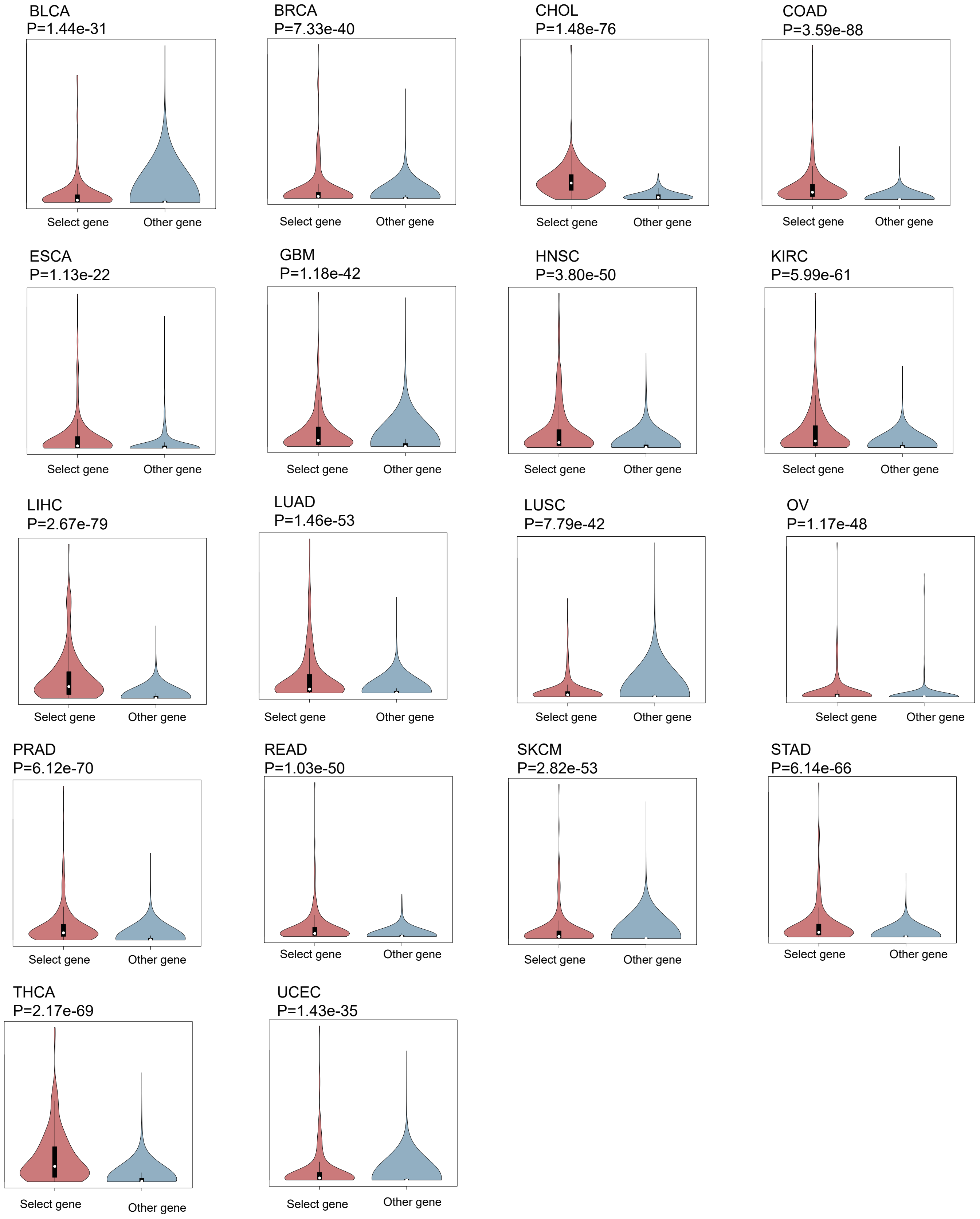

Supplementary Figure 14. The samples are divided into two groups according to tumor purity score, and their partial correlation is calculated, and the ANOVA is performed. Black boxes indicate the interquartile range of the data. White dots indicate the median. Black vertical lines indicate 95% confidence intervals. The width of the violin plot indicates the density of the data.

Supplementary Figure 15

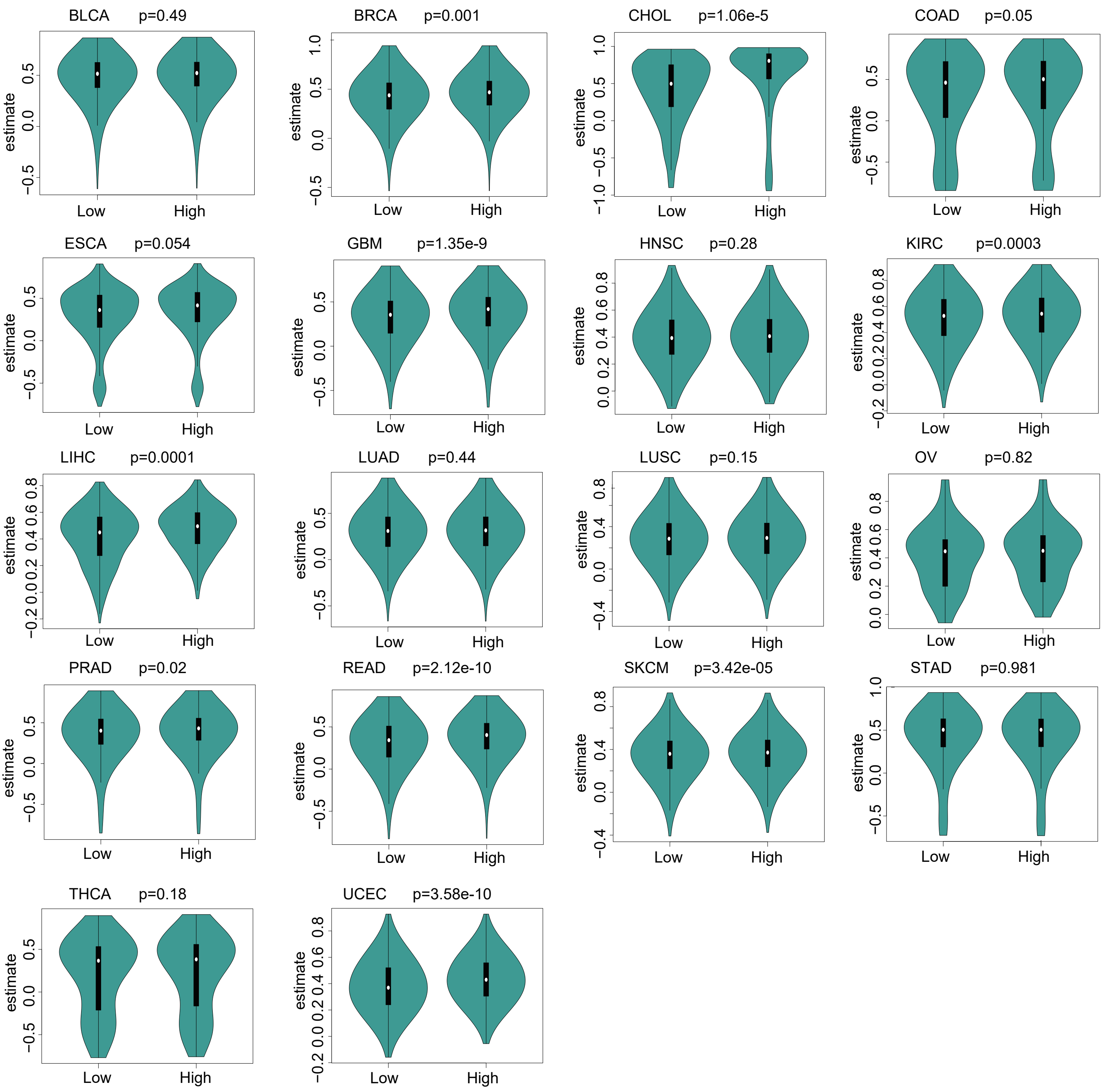

Supplementary Figure 15. Comparison of ICP-associated lnc with immune-associated lnc in ImmLnc work. P-values are calculated using the hypergeometric test with a background gene cunt of 13953. Black boxes indicate the interquartile range of the data. White dots indicate the median. Black vertical lines indicate 95% confidence intervals. The width of the violin plot indicates the density of the data.

Supplementary Table 1. Dataset sources and sample size statistics

TCGA	Detail	Num	GTEx	Num
BLCA	Bladder Urothelial	430		
BRCA	Breast invasive carcinoma	1217		
CHOL	Cholangiocarcinoma	45		
COAD	Colon adenocarcinoma	512		
ESCA	Esophageal carcinoma	173		
GBM	Glioblastoma multiforme	173		
HNSC	Head and Neck squamous cell carcinoma	547		
KIRC	Kidney renal clear cell carcinoma	607		
LIHC	Liver hepatocellular carcinoma	424		
LUAD	Lung adenocarcinoma	585		
LUSC	Lung squamous cell carcinoma	550		
OV	Ovarian serous cystadenocarcinoma	379	Ovary	89
PRAD	Pancreatic adenocarcinoma	551		
READ	Rectum adenocarcinoma	177		
SKCM	Skin Cutaneous Melanoma	472	Skin	234
STAD	Stomach adenocarcinoma	407		
THCA	Thyroid carcinoma	569		
UCEC	Uterine Corpus Endometrial Carcinoma	583		

Supplementary Table 2. Independent dataset for validation

Dataset	Detail	Source
GBM-325	Glioblastoma multiforme	CGGA
PRAD-FR	Pancreatic adenocarcinoma in France	ICGC

Supplementary Table 3. Single-cell datasets

Dataset	Cancer Type	Number of cell
GSE127471	NSCLC	1443
GSE117570	NSCLC	1931
GSE69405	LUAD	208
GSE75688	BRCA	563
GSE118389	BRCA	1534
GSE125449_set2	CHOL	4831
GSE81861	COAD	590
GSE103322	HNSC	5902
GSE125449_set1	LIHC	5115
GSE72056	SKCM	2840

Supplementary Table 4. Dataset used for ICB response analysis

Cohort	Treatment	Sample	Responder	Non-responder	PMID
Gide et al. [1]	anti-CTLA-4+PD-1	32	21	11	30753825
Gide et al. [1]	anti-PD-1	41	19	22	30753825
Van Allen et al. [2]	anti-CTLA-4	37	14	23	26359337
Riaz et al.-Naive [3]	anti-PD-1	25	6	19	29033130
Riaz et al.-Prog [3]	anti-PD-1	26	4	22	29033130

Supplementary References

1 Gide, T. N. , Quek, C. , Menzies, A. M., et al. Distinct Immune Cell Populations Define Response to Anti-PD-1 Monotherapy and Anti-PD-1/Anti-CTLA-4 Combined Therapy. Cancer Cell 35, 238-255 e236. https://www.ncbi.nlm.nih.gov/pubmed/30753825 (2019).

2 Van Allen, E. M. , Miao, D. , Schilling, B., et al. Genomic correlates of response to CTLA-4 blockade in metastatic melanoma. Science 350, 207-211. https://www.ncbi.nlm.nih.gov/pubmed/26359337 (2015).

3 Riaz, N. , Havel, J. J. , Makarov, V., et al. Tumor and Microenvironment Evolution during Immunotherapy with Nivolumab. Cell 171, 934-949 e916. https://www.ncbi.nlm.nih.gov/pubmed/29033130 (2017).
