# Supplementary material for: The landscape of immune checkpoint-related long non-coding RNAs core regulatory circuitry reveals implications for immunoregulation and immunotherapy responses
Source: Commun Biol. 2024 Mar 14;7:327. doi: 10.1038/s42003-024-06004-z (PMC10940638; doi:10.1038/s42003-024-06004-z)
Supplement: Supplementary file 2 — Description of Additional Supplementary Files [file 42003_2024_6004_MOESM2_ESM.pdf]

Description of Additional Supplementary Files

File name: Supplementary Data 1
Description: The source data behind the Figure 1-5 in the paper.

File name: Supplementary Data 2
Description: The source data behind the Figure 6-7 in the paper.
